# The efficacy of ferroptosis-inducing compounds IKE and RSL3 correlates with the expression of ferroptotic pathway regulators CD71 and SLC7A11 in biliary tract cancer cells

**DOI:** 10.1371/journal.pone.0302050

**Published:** 2024-04-11

**Authors:** Dino Bekric, Tobias Kiesslich, Matthias Ocker, Martina Winklmayr, Markus Ritter, Heidemarie Dobias, Marlena Beyreis, Daniel Neureiter, Christian Mayr

**Affiliations:** 1 Center of Physiology, Pathophysiology and Biophysics, Institute of Physiology and Pathophysiology Salzburg, Paracelsus Medical University, Salzburg, Austria; 2 Cancer Cluster Salzburg, Salzburg, Austria; 3 Department of Internal Medicine I, University Clinics Salzburg, Paracelsus Medical University, Salzburg, Austria; 4 Division of Hematology, Oncology, and Cancer Immunology, Medical Department, Charité University Medicine Berlin, Berlin, Germany; 5 Tacalyx GmbH, Berlin, Germany; 6 Ludwig Boltzmann Institute for Arthritis und Rehabilitation, Paracelsus Medical University, Salzburg, Austria; 7 Gastein Research Institute, Paracelsus Medical University, Salzburg, Austria; 8 Kathmandu Medical School of Medical Sciences, Dhulikhel, Nepal; 9 Institute of Pathology, University Clinics Salzburg, Paracelsus Medical University, Salzburg, Austria; Toho University Graduate School of Medicine, JAPAN

## Abstract

**Introduction:**

Biliary tract cancer (BTC) is a lethal disease with a bad overall survivability, partly arising from inadequate therapeutic alternatives, detection at a belated stage, and a resistance to common therapeutic approaches. Ferroptosis is a form of programmed cell death that depends on reactive oxygen species (ROS) and iron, causing excessive peroxidation of polyunsaturated fatty acids (PUFAs). Therefore, the objective of this investigation is, whether ferroptosis can be induced in BTC in vitro and whether this induction is dependent on specific molecular markers.

**Methods:**

The study conducted resazurin assay and IC_25/50_ calculation to explore the possible cytotoxic outcomes of different classes of ferroptosis-inducing substances (FINs) on a comprehensive in vitro model of 11 BTC cell lines. Combinatory treatments with different cell death inhibitors were performed to evaluate the magnitude of ferroptosis induction. To ascertain whether ferroptotic cell death occurred, liperfluo and iron assay kits were employed to evaluate lipid ROS and intracellular iron abundance. Potential biomarkers of ferroptosis sensitivity were then assessed via western blot analysis, a rtPCR panel and functional assay kits.

**Results:**

The study found that different FINs reduced cell viability in a cell line-dependent manner. In addition, we measured increased lipid ROS and intracellular Fe^2+^ levels upon exposure to FINs in BTC cells. Combining FINs with inhibitors of ferroptosis, necroptosis or apoptosis suggests the occurrence of ferroptotic events in BTC cell lines CCC-5, HuH-28 and KKU-055. Furthermore, we found that BTC cells display a heterogeneous profile regarding different molecular genes/markers of ferroptosis. Subsequent analysis revealed that sensitivity of BTC cells towards IKE and RSL3 positively correlated with CD71 and SLC7A11 protein expression.

**Conclusion:**

Our results demonstrate that induction of ferroptosis is a promising approach to inhibit BTC cell growth and that the sensitivity of BTC cells towards ferroptosis induction might be dependent on molecular markers such as CD71 and SLC7A11.

## Introduction

Biliary tract cancer (BTC) comprises intra- and extrahepatic cholangiocarcinoma (CCA) and gallbladder cancer (GBC) and is associated with a dismal outcome and a very poor five-year survival rate. The incidence rate varies by geographic region. In Eastern regions of the world, the incidence stands at 60 per 100,000 population, whereas in the Western World, the rate ranges from 0.5 to 2 per 100,000 population [1].

Reasons for that include diagnosis at an already mid—to advanced stage and currently ineffective therapeutic strategies [1, 2]. The current treatment options for BTC consist of surgery when localized or combined chemotherapy (cisplatin, gemcitabine) when locally advanced and non-resectable [3, 4]. Molecular profiling might identify patients eligible for targeted therapies (IDH1/2 mutations, FGFR 1–3 mutations and fusions, BRAF mutation, mismatch repair (MMR) deficiency, HER2 overexpression, NTRK fusions) but this applies only to selected patients [5]. Current research on BTC suggests potential alternative therapeutic approaches, including EZH2 and HDACs inhibition, but these are currently only at the preclinical stage [6, 7]. Therefore, with the poor overall survival rate and the shortfall of effective treatment options, the discovery of novel therapeutic options against BTC is of utmost importance.

In 2012, Dixon et al. described a new form of regulated cell death, called ferroptosis [8]. This type of cell death is iron- and reactive oxygen species (ROS)-dependent and can be distinguished from the other “classical” forms of regulated cell death, such as apoptosis and necroptosis in a morphological and mechanistical manner [9]. The main characteristic of ferroptosis is the generation of lipid ROS. Lipid ROS are generated by peroxidation of polyunsaturated fatty acids (PUFAs) and a massive accumulation of iron and are eventually responsible for membrane rupture [9].

Regarding the role of ferroptosis in cancer, recent studies have shown that induction of ferroptosis is a potential anti-cancer approach [10]. Cancer cells might be particularly vulnerable towards ferroptosis induction as their specific metabolism and aberrant proliferation result in an increased iron demand compared to non-cancerous cells—a phenomenon termed ‘iron addiction’ [11, 12]. Additionally, oxidative stress levels are elevated in cancer cells, which is compensated by the upregulation of antioxidant factors like ferroptosis suppressor protein-1 (FSP-1), solute carrier family 7 member 11 (SLC7A11) and glutathione peroxidase 4 (GPX4) [11–13]. These factors are important negative regulators of ferroptosis as they all counteract the accumulation of lipid-ROS via different molecular functions: SLC7A11 for examples is a cystine/glutamate antiporter that is important for the uptake of cysteine, which is then converted into cysteine and required for the synthesis of glutathione (GSH) [14]. GSH serves as a cofactor for GPX4, an enzyme that protects cells from lipid peroxidation via converting the toxic lipid peroxides into nontoxic lipid alcohols [14]. FSP-1 on the other hand is a NADP(H)-ubiquinone oxidoreductase that functions as a glutathione independent inhibitor of phospholipid peroxidation via the reduction of ubiquinone to ubiquinol [14, 15]. The strong reliance of cancer cells on these antioxidant factors to counteract their intrinsically elevated ROS might render them vulnerable to ferroptosis induction by specific inhibition of these factors [11, 12]. Therefore, the induction of ferroptosis in cancer cells depends on two main factors: the elevation of intracellular iron levels and impairment of the cell’s antioxidant protection systems [11, 12]. Based on this rationale, class I ferroptosis inducers (FINs) such as erastin target SLC7A11, whereas class II FINs such as RSL-3 inhibit GPX4 activity [9]. Class III FINs like as FIN56 also target GPX4 and lead to its degradation [9]. Lastly, class IV FINs such as FINO2 modify the labile iron pool and indirectly inhibit GPX4 [9]. Current studies suggest that treatment of cancer cells with different classes of established FINs inhibits cell viability and tumor growth in vitro and in vivo in several tumor types: for instance, Zhao et al. could demonstrate that RSL-3 reduced the cell viability of hepatocellular carcinoma cells [16]. Likewise, in diffuse large B cell lymphoma cells, Yang and coworkers showed that treatment with erastin and RSL3 increased intracellular ROS levels and attenuated cell proliferation and that this effect could be reversed by the application of the antioxidant Vitamin E [17].

Since ferroptosis is a non-apoptotic but regulated and therefore targetable form of cell death, induction of ferroptosis represents a specifically attractive therapeutic approach for cancer entities such as BTC that develop resistance against apoptosis-inducing chemotherapeutics (e.g. cisplatin) [3, 4]. Currently, there is some evidence that ferroptosis can be induced in BTC cells: Li et al. demonstrated that lithocholic acid induces ferroptosis in GBC cell lines by reducing GSH/GSSG and NADP(H) ratios and elevating ROS levels [18]. Furthermore, tumor growth in GBC xenograft models was impaired when treated with lithocholic acid [18]. In another study by Wang and coworkers, isoliquiritigenin (ISL), a natural compound from the licorice roots, led to elevated iron, ROS and lipid peroxide levels and a reduced GSH/GSSG ratio–all hallmarks of ferroptosis—accompanied by inhibition of proliferation in GBC cells [19]. Furthermore, GPX4 was downregulated in vitro and in vivo following ISL treatment [19]. Additionally, further studies revealed the regulation of ferroptosis in BTC by long non-coding RNAs, transcription factors, epigenetic modulators and other functional proteins [20–24]. For example, transcription factor AP-2 alpha (TFAP2A) was shown to inhibit ferroptosis [25]. In GBC cells, TFAP2A was overexpressed and its inhibition led to a reduced migration, invasion and proliferation as well as to increased intracellular Fe^2+^ and MDA levels, again typical characteristics of ferroptosis induction [25].

Initial studies suggested induction of ferroptosis as a potential novel strategy against BTC, but definitive data on ferroptosis induction in BTC are still sparse. In the current study, we therefore aimed for a comprehensive investigation of the potential of ferroptosis induction in a BTC in vitro model. Specifically, we evaluated the effect of different ferroptosis inducers comprising members of all four FIN classes on cell viability and ferroptosis-related factors. Following proof of ferroptosis induction by several independent approaches, moreover, we aimed to identify markers that might predict the sensitivity of BTC cells towards ferroptosis induction.

## Materials and methods

### Cell culture and substances

The hepatocellular carcinoma cell line Hep-G2 (ECACC 85011430) was purchased from the European Collection of Authenticated Cell Cultures (ECACC, Salisbury, United Kingdom). Cervix carcinoma cell line HeLa (ACC 57) was purchased from German Collection of Microorganisms and Cell Cultures GmbH (DSMZ; Braunschweig, Germany). BTC cell lines CCC-5 (ACC 810, RRID: CVCL_LM83 [26]), EGi-1 (ACC 385, RRID: CVCL_1193, [27]) and TFK-1 (ACC 344, RRID:CVCL_2214, [28]) were purchased from DSMZ. BTC cell lines HuH-28 (JCRB0426, RRID: CVCL_2955), HuCCT-1 (JCRB0425, RRID: CVCL_0324, [29]), KKU-055 (JCRB1551, RRID: CVCL_M258), KKU-100 (JCRB1568, RRID: CVCL_3996, [30]), NOZ (JCRB1033, RRID: CVCL_3079, [31]), OCUG-1 (JCRB0191, RRID: CVCL_3083, [32]) and OZ (JCRB1032, RRID: CVCL_3118, [33]) were obtained from the Japanese Collection of Research Bioresources Cell Bank (JCRB, Osaka, Japan). The non-tumor cholangiocyte cell line MMNK-1 was purchased from JCRB (JCRB1554, RRID: CVCL_M266). Cells were cultured in a cell incubator at 37°C, 5% CO_2_ and humidified atmosphere using either a Roswell Park Memorial Institute (RPMI)-1640 medium or high-glucose Dulbecco’s modified Eagle’s medium (DMEM), all purchased from ThermoFisher Scientific/Gibco (Waltham, MA, USA). Media were supplemented with 10% (v/v) fetal bovine serum (FBS, Biochrom, Berlin, Germany), 1% antibiotic-antimycotic (ABAM, Merck, Darmstadt, Germany), 1 nM sodium pyruvate (Pan Biotech, Aidenbach, Germany) and 10 mM HEPES (Pan Biotech). For 96-well microplates, 6-well plates and 100 mm dishes, 10,000, 1*10^5^ and 2*10^6^ cells were seeded, respectively and let grown over night. For washing steps, Dulbecco’s Phosphate Buffered Saline (DPBS; Pan Biotech) was used. FINs stocks were prepared in dimethyl sulfoxide (DMSO; Sigma Aldrich) and stored as aliquots at -20°C and -80°C (Table 1) Cell death inhibitors were purchased as solved stock solutions and stored as aliquots at -80°C. (Table 1).

**Table 1:** Ferroptosis inducers and cell death inhibitors.

	Substance	Mechanism (FIN class or target)	Stock, mM	Cat.-No.
Ferroptosis inducer	IKE	System xCT Inhibition (I)	5.0	MCE-HY-114481^a^
	RSL3	GPX4 Inhibition (II)	5.0	MCE-HY-100218A^a^
	FIN56	GPX4 Degradation (III)	5.0	MCE-HY-103087^a^
	FINO2	GPX4 inhibition, Iron oxidation (IV)	5.0	869298-31-7^b^
	Brequinar	DHODH Inhibition (n.a.)	1.0	S6626^c^
	iFSP1	FSP1 Inhibition (n.a.)	5.0	150651-39-1^b^
Cell Death Inhibitors	DFX	Ferroptosis inhibitor (iron chelator)	10.0	MCE-HY-B0988^a^
	Ferrostatin-1	Ferroptosis inhibitor (antioxidant)	10.0	MCE-HY-100579^a^
	Necrostatin-1	Necroptosis inhibitor (RIP1 kinase)	10.0	MCE-HY-15760^a^
	Z-VAD-FMK	Apoptosis inhibitor (pan-caspase)	10.0	MCE-HY-16658B^a^

^a^…MedChemExpress (Monmouth Junction, NJ, USA)

^b^…Cayman Chemicals (Michigan, MI, USA)

^c^…Selleckchem (Houston, TX, USA)

Abbreviations: DFX = deferoxamine (mesylate), n.a. = not applicable.

### Cell viability

After seeding in 96-well microplates, cells were washed with serum-free DMEM (sfDMEM) and incubated with a 10-step 1:2 dilution series of Brequinar FINO2, RSL3 (10 μM- 0.02 μM), FIN56 (40 μM– 0.08 μM), iFSP1 and IKE (50 μM– 0.1 μM) in serum-free DMEM for 48 h. Cell viability was then measured via resazurin assay based on a fluorescence readout (exc: 535 nm; em: 585 nm) on a Spark multimode reader (Tecan, Grödig, Austria) as described [34]. IC_25/50_ values were calculated via four-parameter logistic regression.

For evaluation of the cytotoxic effect combined treatment with FINs and cell death inhibitors, CCC-5, HuH-28 and KKU-055 as well as Hep-G2 and HeLa cells were seeded, washed once with sfDMEM and incubated with FINs and cell death inhibitors only or a combination FINs and cell death inhibitors for 24 h in sfDMEM, followed by resazurin-based viability assay. Regarding the used concentrations, the highest concentrations of the respective dilution series were used for the different FINs, whereas for deferoxamine mesylate, ferrostatin-1, necrostatin-1 and Z-VAD-FMK, 20 μM were used.

### Protein expression

After seeding in 100 mm dishes, cells were washed with DBPS, harvested with trypsin-EDTA, centrifuged (400 g, 5 min), counted and stored as cell pellets at -20°C. For further processing, cell pellets were thawed, resuspended in DPBS to a concentration of 10^7^ cells per ml and lysed via sonication with a Sonopuls HD70 (UW 70 ultrasound head, Bandelin; 10 pulses). The samples were then centrifuged (17,000 x g, 10 min at 4°C) and the supernatant was mixed with one volume of 2X sodium dodecyl sulfate (SDS) (Thermo Fisher Scientific, Waltham, MA, USA), incubated for 5 min at 95°C and centrifuged (400 x g, 5 min at room temperature). Proteins were then separated on gradient SDS gels (4–20% Mini-PROTEAN gels, Biorad, Hercules, CA, USA), where each slot contained the volume equivalent of 100,000 cells for 90 min at 100 V. The gels were then transferred to nitrocellulose membranes (Biorad) and blotted using Trans-Blot Turbo System (7 min, 25 V; Biorad). Membranes were incubated for one hour with Blotting Grade Blocker (Biorad) to prevent unspecific binding, following incubation with primary antibodies overnight at 4°C (see Table 2). Blots were then washed with TBS-T and incubated for 1 h at room temperature with the respective secondary antibodies (see Table 2). For detection of proteins, blots were incubated for two minutes with the Signal Fire ECL Reagent (Cell Signaling Technologies). Chemiluminescence was analyzed using a ChemiDoc MP System and the Image Lab Software (Biorad). Grey densities of bands were calculated via ImageJ (V1.54d; NIH, Bethesda, MD, USA) to evaluate the protein expression related to the loading control β-actin.

**Table 2:** Antibodies used for western blot analyses.

Primary Antibody	Dilution	Cat.-No.	Source
Anti-ACSL4	1:500	sc-365230^a^	mouse
Anti-CD71	1:500	sc-32272^a^	mouse
Anti-ferritin heavy chain	1:500	sc-376594^a^	mouse
Anti-GPX-4	1:500	sc-166570^a^	mouse
Anti-β-actin	1:2000	4970^b^	rabbit
Anti-xCT	1:1000	ab175186^c^	rabbit
**Secondary Antibody**			
Anti-rabbit-IgG HRP-linked	1:2000	7074^b^	goat
Anti-mouse-IgGк BP-HRP	1:2000	sc-516102^a^	mouse

Vendors

^a^…Santa Cruz Biotechnology (Dallas, TX, USA)

^b^…Cell signaling Technology (Danvers, MA, USA)

^c^…Abcam (Cambridge, United Kingdom)

### Lipid ROS

For the Lipid Peroxide Detection kit (Dojindo Laboratories, Kumamoto, Japan), the BTC cell lines CCC-5, HuH-28 and KKU-055 as well as non-BTC cell lines HeLa and Hep-G2 were seeded in 6-well plates and let grown overnight. Cells were washed with sfDMEM and incubated with 3 μM Liperfluo in Fluorobrite DMEM (Thermofisher Scientific). Afterwards, cells were washed with DPBS and incubated with FINs using the highest concentrations of the respective dilution series for 6 h. Treatment of cells with 500 μM tert-butyl hydroperoxide (Sigma Aldrich, St. Louis, MO, USA) for 1.5 h was used as a positive control. Cells were washed again with DBPS and lysed using 100 μl DMSO for 10 min at room temperature. The lysate was mixed by pipetting and transferred to a black-walled 96-well microplate and lipid peroxidation was assessed via the fluorescence of Liperfluo (λ_ex_ = 488 nm; λ_em_ = 550 nm) on a via Spark multimode reader.

### Intracellular iron

To determine intracellular Fe^2+^ levels at baseline (all BTC cell lines and Hep-G2, and HeLa) or after 24 h FIN treatment (for CCC-5, HuH-28, KKU-055), the Iron Assay Kit (Sigma Aldrich) was used. Cells were seeded in 100 mm dishes, let grown overnight and directly assayed or incubated with FINs for 24 h. Afterwards, cells were washed with DBPS, harvested with trypsin/EDTA and the Fe^2+^ content was measured colorimetrically at 593 nm via a Spark multimode reader according to the manufacturer’s protocol.

### GPX-activity

For the Glutathione Peroxidase Assay Kit (Cayman Chemical), all BTC cell lines as well as Hep-G2 and HeLa were seeded in 100 mm dishes and let grown overnight. Cells were then washed with DPBS and harvested with a rubber policeman. GPX-activity was then measured colorimetrically at 340 nm with a Spark multimode reader according to the manufacturer’s protocol.

### Total NADP(H)

For the Total NADP and NADPH Assay Kit (Abcam), all BTC cell lines as well as Hep-G2 and HeLa were seeded in 100 mm dishes and let grown overnight. Cells were then washed with DPBS, harvested with trypsin/EDTA and total NADPH/NADH was measured according to the manufacturer’s protocol on a Spark multimode reader colorimetrically at 460 nm.

### Caspase 3/7 activity assay

CCC-5, HuH-28 and KKU-055 cells were seeded in transparent 96-well microplates and let grown overnight. Cells were then washed with sfDMEM and incubated with different FINs using the highest concentration of the respective dilution series for 24 h. Staurosporine (Selleckchem) was used as a positive control (5 μM). Caspase 3/7 activity was measured on a Spark multimode reader using a Caspase-Glo®3/7Assay (Promega, Madison, WI, USA) according to the manufacturer’s protocol.

### mRNA expression

BTC cell lines were seeded in 60 mm dishes, grown overnight and washed once with DBPS. Total RNA was isolated via TRI Reagent (Merck, Rahway, NH, USA) and the Direct-zol RNA Miniprep kit (Zymo Research, Irvine, CA, USA) according to manufacturers’ protocol. cDNA synthesis was done via GoScript Reverse Transcriptase kit (Promega) and Real Time PCR was performed on a ViiA7 real-time PCR system (Applied Biosystems, Thermo Fisher Scientific) using the GoTaq® Master Mix (SYBR Green, Promega). The Human Ferroptosis PCR Primer Library (HFER-1) probing for 88 ferroptosis-related and eight housekeeping genes was obtained from RealTime Primers (obtained from Biomol, Hamburg, Germany). Primers were dissolved in 40 μl H_2_O to obtain 10 μM stocks, stored at -20°C and used in final concentration of 0.6 μM.

Using the default setting of QuantStudio Real-Time PCR Software (v. 1.6.1, AppliedBiosystems), individual wells were excluded from further analysis, in case of i) low passive reference signal, (ii) exponential algorithm failed, and (iii) if identified as outlier. For all genes, the Ct threshold was manually set to 0.04 and data were further processed in Microsoft Excel (Redmond, WA, USA). For comparison of mRNA expression across cell lines, Ct values were normalized (2^-ΔCt^) using multiple referencing with ATPB, B2M, GAPD and GUSB as those housekeeping genes that met additional quality criteria (evaluable expression in all cell lines, mean Ct <40, standard deviation <3, and ≥2 evaluable technical replicates). Target genes were excluded from analysis if mean Ct ≥40, standard deviation >3, and <2 evaluable technical replicates.

### In silico analysis

An additional bioinformatic query was performed on the online database OncoDB (http://www.oncodb.org/; last accessed 2023-10-10 [35]) University of Alabama at Birmingham CANcer data (UALCAN) analysis portal (http://ualcan.path.uab.edu/; last accessed 2023-10-10 [36]) using data from The Cancer Genome Atlas (TCGA) project [37] and STRING (https://string-db.org/; last accessed 2023-10-10 [38]). OncoDB was used to evaluate the gene expression pattern of CD71 (gene name TFRC) and SLC7A11 in BTC in relation to clinico-pathological parameters. Possible protein-protein interactions were also investigated using STRING.

### Statistics

If not otherwise stated, all presented data points represent mean values of at least three independent biological replicates ± standard error of the mean (SEM) and each biological replicate consisted of an appropriate number of technical replicates. To test for significant differences between untreated control (UTC) and treated samples, unpaired Student’s t-test as well as ANOVA test with Bonferroni correction were applied. Pearson’s correlation analysis was employed to investigate the association between FINs’ IC_50_ / IC_25_ values and ferroptosis-related markers. Data fits, statistical calculations and graphing were done using OriginPro 2023b (OriginLab, Northhampton, MA, USA). Statistical results were considered significant p < 0.05 (*) or highly significant p < 0.01 (**).

## Results

### Ferroptosis inducers reduce cell viability of BTC cells

**Fig 1:**
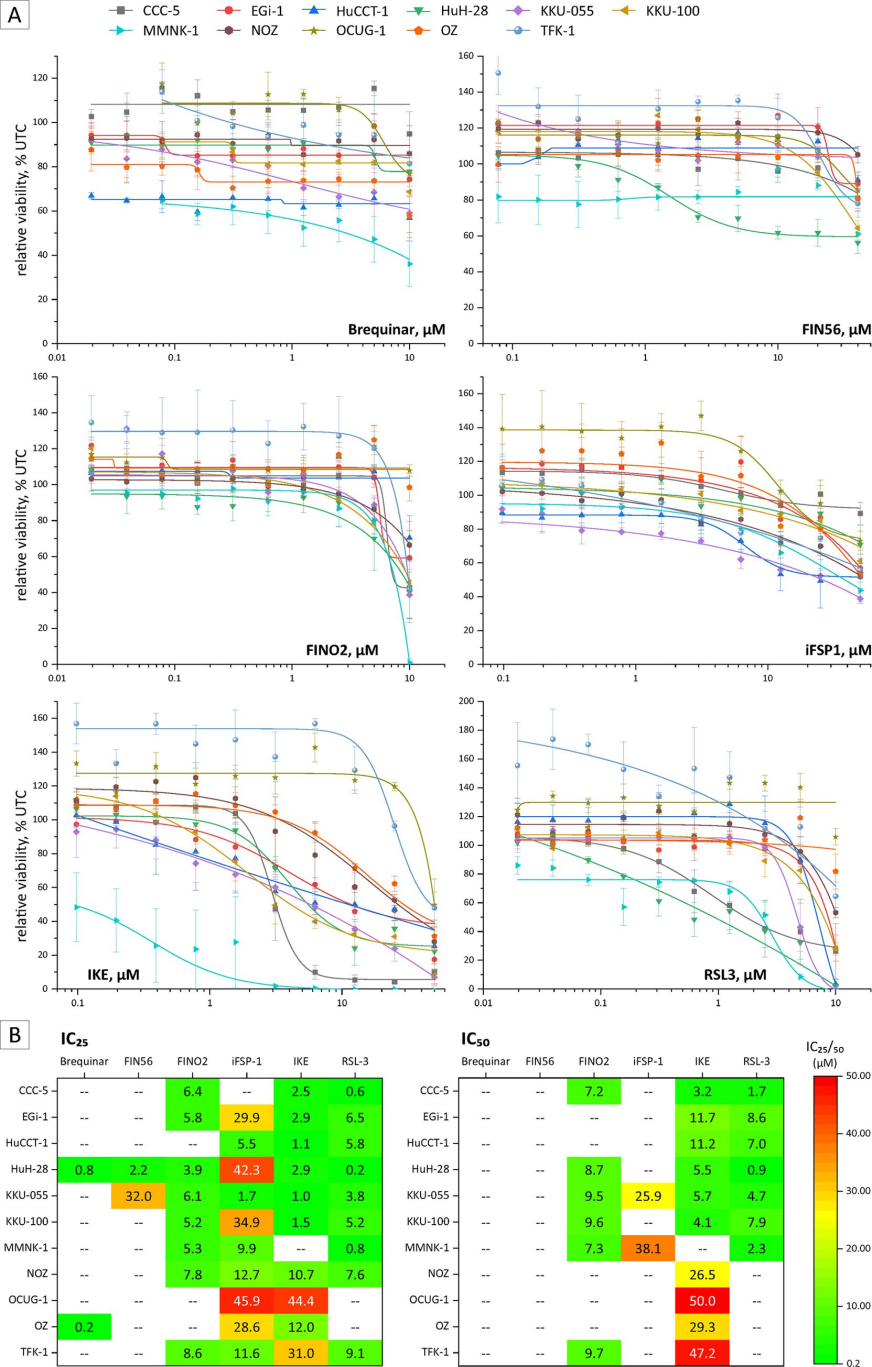
Cell viability is attenuated in a cell line—and substance—specific manner in BTC following FINs treatment. (A) Cell viability of 10 BTC cell lines and non-cholangiocyte cell line MMNK-1 following a 10-step 1:2 dilution series starting with 10 μM brequinar, 40 μM FIN56, 10 μM FINO2, 50 μM iFSP1, 50 μM IKE and 10 μM RSL3 treatment for 48 h; Data is related to untreated control cells (UTC) and shown as mean values +/- SEM of n = 3 biological replicates. (B) Heatmap of calculated IC25/50 values (via four-parameter logistic expression) of FINs in BTC cell lines.

To assess the cytotoxicity of FINs in BTC cell lines, n = 10 BTC cell lines and non-tumor cholangiocytes (MMNK-1) were treated with different concentrations of FINs for 48 h, and cell viability was measured using the resazurin assay. As shown in Fig 1A, treatment of BTC cells with FINO2, IKE, and RSL3 resulted in a clear dose-dependent reduction of cell viability, whereas the cells were less sensitive towards treatment with brequinar, FIN56 and iFSP1. HeLa and HepG2 were used as cell lines known to be sensitive towards FINs [39, 40]; see S1 Fig. The heterogeneous response of BTC cells is also reflected by the corresponding IC_25_ and IC_50_ values (Fig 1B). Due to their highest sensitivity towards FIN treatment, BTC cell lines CCC-5, HuH-28, and KKU-055 were selected for further experiments, including microscopic evaluation of cell morphology, confirming the observed reduction of cell viability (see S2 Fig).

### Cell death induced by ferroptosis inducers is partially reversible by deferoxamine mesylate and ferrostatin-1

Although FINs are postulated to induce ferroptosis, these drugs can also induce alternative forms of cell death [41]. Therefore, we evaluated in the next step whether the observed reduction of cell viability is (partially) caused by induction of ferroptosis.

**Fig 2:**
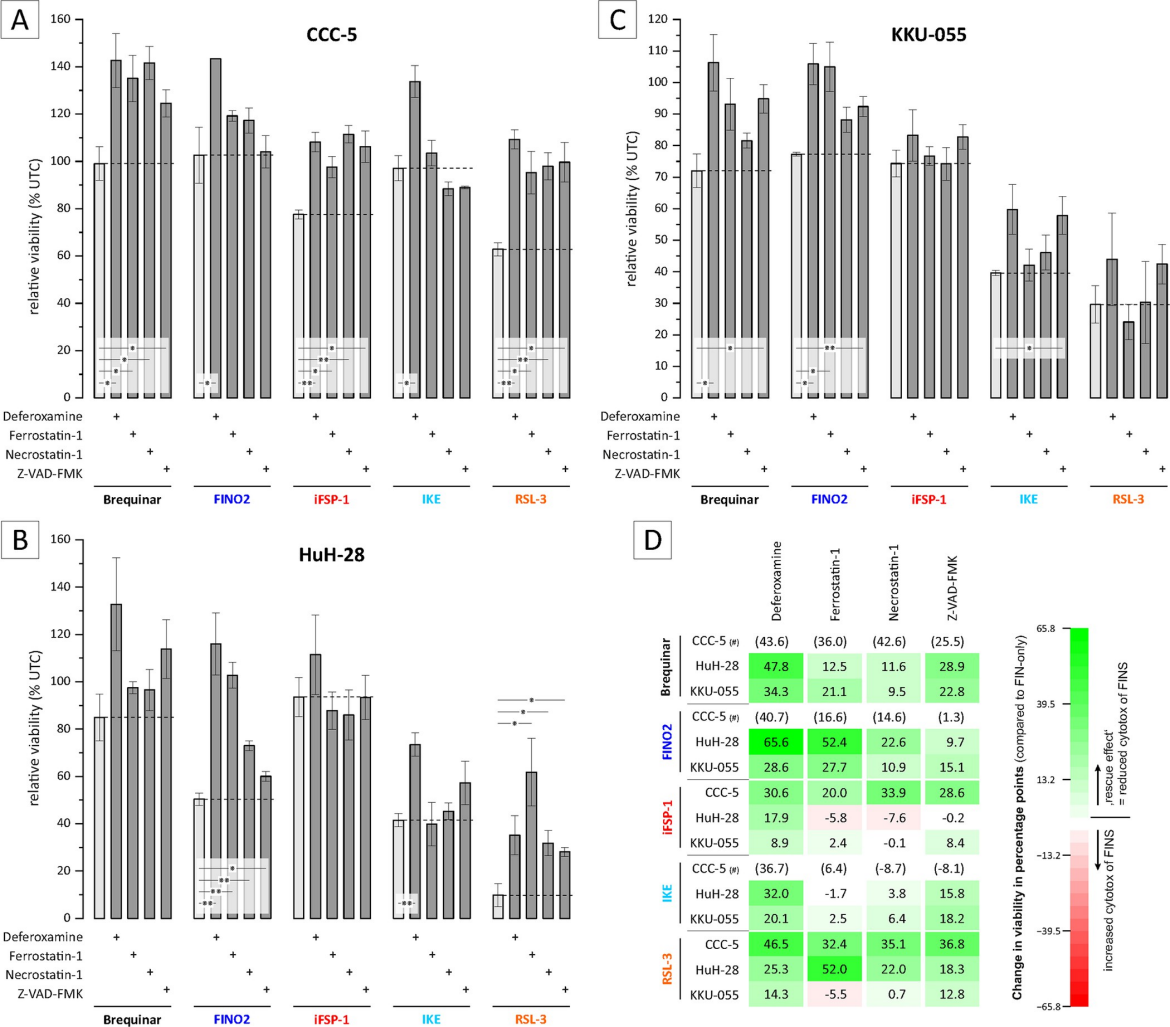
Reduction of cell viability via FINs is (partially) reversed by deferoxamine mesylate and ferrostatin-1. Cells were treated with 1 μM brequinar, 10 μM FINO2, 50 μM iFSP1, 50 μM IKE and 10 μM RSL3 with 20 μM of deferoxamine mesylate, ferrostatin-1, necrostatin-1 and Z-VAD-FMK. (A) Cell viability data is shown as mean values +/- SEM of n = 3 biological replicates of CCC-5 cells with FINs (light grey) and FINS + cell death inhibitors (dark grey) treatment for 24 h. (B) Cell viability data is shown as mean values +/- SEM of n = 3 biological replicates of HuH-28 cells with FINs (light grey) and FINS + cell death inhibitors (dark grey) for 24 h. (C) Cell viability data is shown as mean values +/- SEM of n = 3 biological replicates of KKU-055 cells with FINs (light grey) and FINS + cell death inhibitors (dark grey) for 24 h. (D) The effect of the different cell death inhibitors on the FIN-induced cell death was expressed by the positive/negative difference (Delta% Fig 2A–2C) related to FIN-only (green: “rescue effect”; red: viability is further decreased) * = significant p < 0.05, ** = highly significant p < 0.01; UTC = untreated control # = these values don’t represent a definitive ‘rescue effect’ since treatment with these FINS alone did not result in a decrease of cell viability.

Since ferroptosis is an iron- and ROS-dependent process, addition of the iron chelator deferoxamine mesylate and/or the antioxidant ferrostatin-1 to FIN-treated cells should lead to a (partial) reversal of the cytotoxic effect of FINs, whereas addition of the apoptosis inhibitor Z-VAD-FMK and the necroptosis inhibitor necrostatin-1 should not affect ferroptosis-based reduction in cell viability [9]. We therefore combined different FINs (except FIN56, which had no effect on BTC cell viability) with deferoxamine mesylate, ferrostatin-1, necrostatin-1 and Z-VAD-FMK (HeLa and Hep-G2 were used as cell lines that are known to be sensitive towards treatment with FINs–S3A and S3B Fig; cell death inhibitors only had no effect on cell viability–S3C Fig). As shown in Fig 2A–2C, addition of deferoxamine mesylate as well as ferrostatin-1 resulted in (significant) partial reversal of the cytotoxic effects of FINs in a substance- and cell line-dependent manner. Although we also observed increased cell viability when FINs were combined with necrostatin-1 and Z-VAD-FMK, respectively, the reversal of the cytotoxicity induced by FINs was more pronounced when cells were co-treated with deferoxamine mesylate and ferrostatin-1, respectively (Fig 2D).

### Ferroptosis inducers increase Fe^2+^ and lipid ROS levels in BTC cells

**Fig 3:**
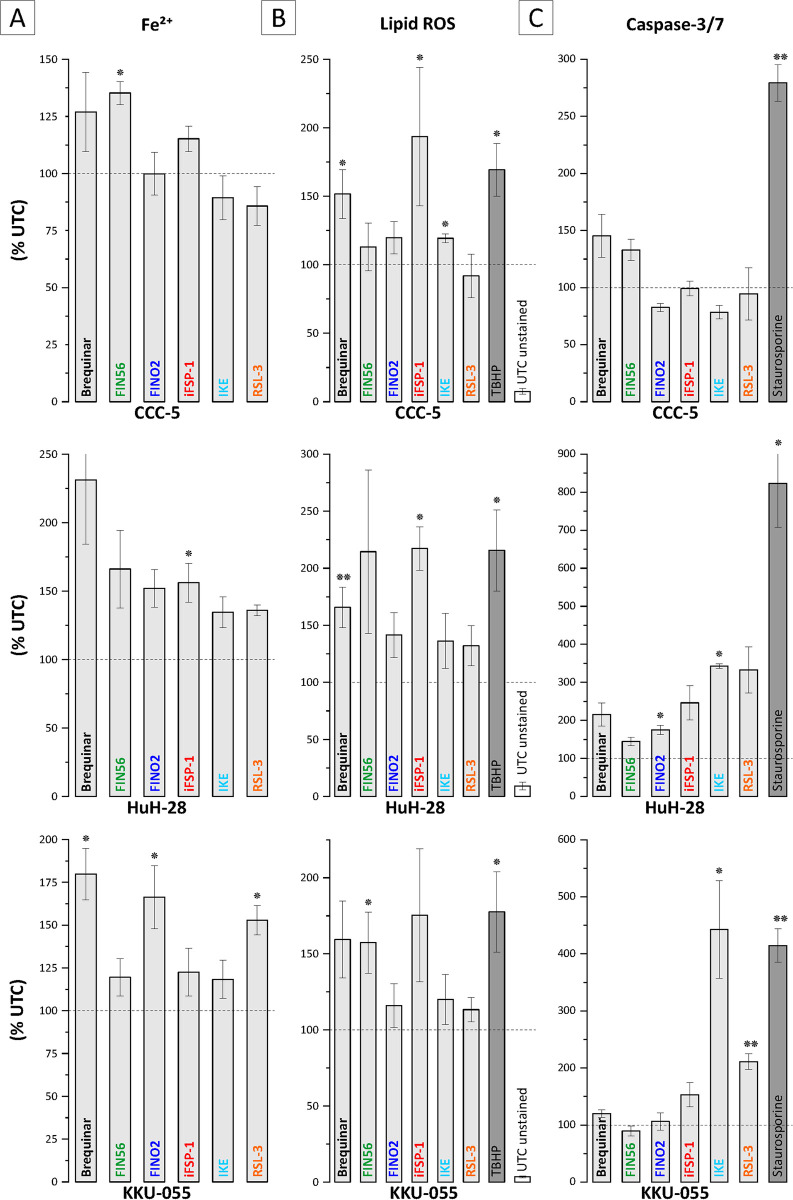
Fe^2+^ and lipid ROS levels are elevated following FIN treatment in selected BTC cell lines. Cells were treated with 1 μM brequinar, 10 μM FINO2, 50 μM iFSP1, 50 μM IKE and 10 μM RSL3. (A) Intracellular iron levels after FIN treatment for 24 h in CCC-5, HuH-28 and KKU-055 cells. Data is related to untreated control cells and shown as mean +/- SEM of n = 3 biological replicates. (B) Lipid ROS levels after FIN treatment for 24 h in CCC-5, HuH-28 and KKU-055 cells. Data is related to untreated control cells and shown as mean +/- SEM of n = 3 biological replicates. (C) Caspase 3/7 activity after FIN treatment for 24 h in CCC-5, HuH-28 and KKU-055 cells. Data is related to untreated control cells and shown as mean +/- SEM of n = 3 biological replicates. * = significant p < 0.05, ** highly significant p < 0.01; UTC = untreated control.

Induction of ferroptosis is also characterized by increases in intracellular Fe^2+^ and lipid ROS levels [9]. In dependence of the used FIN, treatment of BTC cell lines CCC-5, HuH-28, and KKU-055 for 24 h resulted in an increase of both, Fe^2+^ levels and lipid ROS (Fig 3A and 3B). We additionally measured caspase-3/7 activity to evaluate the contribution of apoptosis to the observed decline in cell viability induced by FINs. As displayed in Fig 3C, we measured an increase in caspase-3/7 activity, although the magnitude of increase was low compared to the positive control staurosporine (except for IKE in KKU-055 cells), generally suggesting a rather small contribution of apoptosis induction.

### Ferroptosis-related genes and markers correlate with sensitivity towards ferroptosis inducers

After observing that selected FINs decrease cell viability in a cell line-specific manner involving ferroptosis, we next asked whether the sensitivity of BTC cells towards FINs can be associated with molecular markers and/or the expression of specific genes. We therefore correlated the IC_25_ and IC_50_ values of RSL3, IKE, FINO2 and iFSP1 (not calculable for brequinar and FIN56) with factors relevant to ferroptosis (GPX activity, NADP(H) level, intracellular Fe^2+^, ACSL4, CD71, FTH1, GPX4 and xCT protein expression) as well as with the mRNA expression of a panel of n = 88 ferroptosis-related genes.

**Fig 4:**
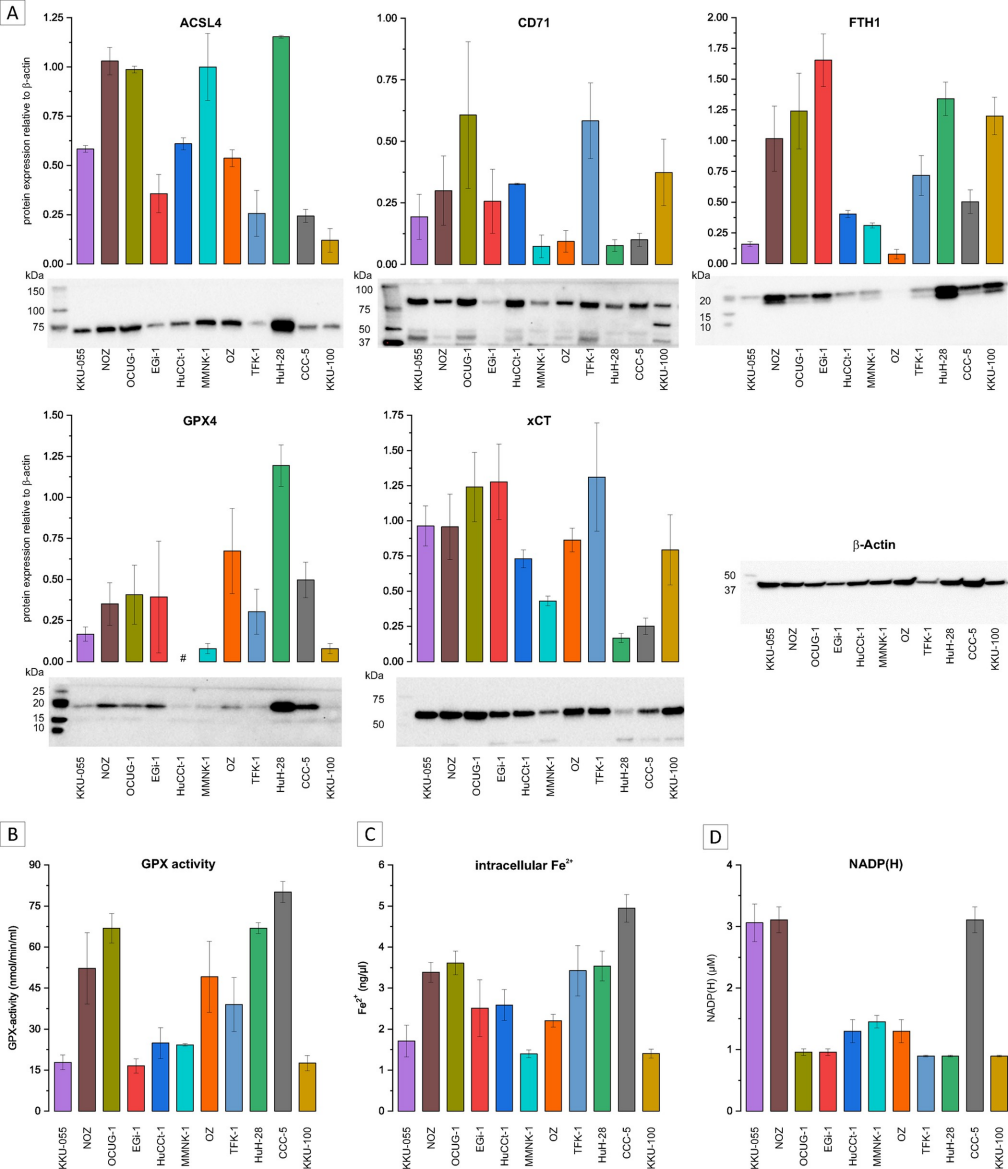
Baseline protein expression of ACSL4, CD71, FTH1, GPX4 and xCT; GPX-activity, intracellular Fe^2+^ levels and NADP(H) concentration in BTC cell lines. (A) Baseline protein expression of ACSL4, CD71, FTH1, GPX4 and xCT in BTC cells with representative western blot images. Shown are data as mean values +/- SEM of n = 3 biological replicates relative to β-actin expression. (B) Baseline GPX-activity of BTC cells. Data is shown as mean +/- SEM of n = 3 biological replicates. (C) Baseline intracellular Fe^2+^ levels of BTC cells. Data is shown as mean +/- SEM of n = 3 biological replicates (D) Baseline NADP(H) concentration in BTC cells. Data is shown as mean +/- SEM of n = 3 biological replicates; # = not quantifiable in all 3 biological replicates.

As shown in Fig 4, (and S4 Fig for Hela and Hep-G2), the examination of pertinent ferroptosis markers in BTC cell lines exposed a heterogeneous profile across BTC Cell lines. Interestingly, the three most FIN-sensitive cell lines (CCC-5, HuH-28 and KKU-055) did not notably differ from the remaining cell lines (see Fig 4). Noteworthy, in BTC cell line HuCCt-1, GPX4 protein expression could not be quantified (see Fig 4A).

The complete results of mRNA analyses for the panel of 88 ferroptosis-related factors is shown in S5 Fig. The following genes could not be detected in any of the analyzed BTC cell lines: BBC3, HAMP, LOX, MAP1LC3A, NFE2L2, NOX4, PCBP1, SLC11A2, SLC3A2 and TFR2; in addition, CS, CHAC1, HMOX2 were excluded because of amplification in the non- template control.

**Fig 5:**
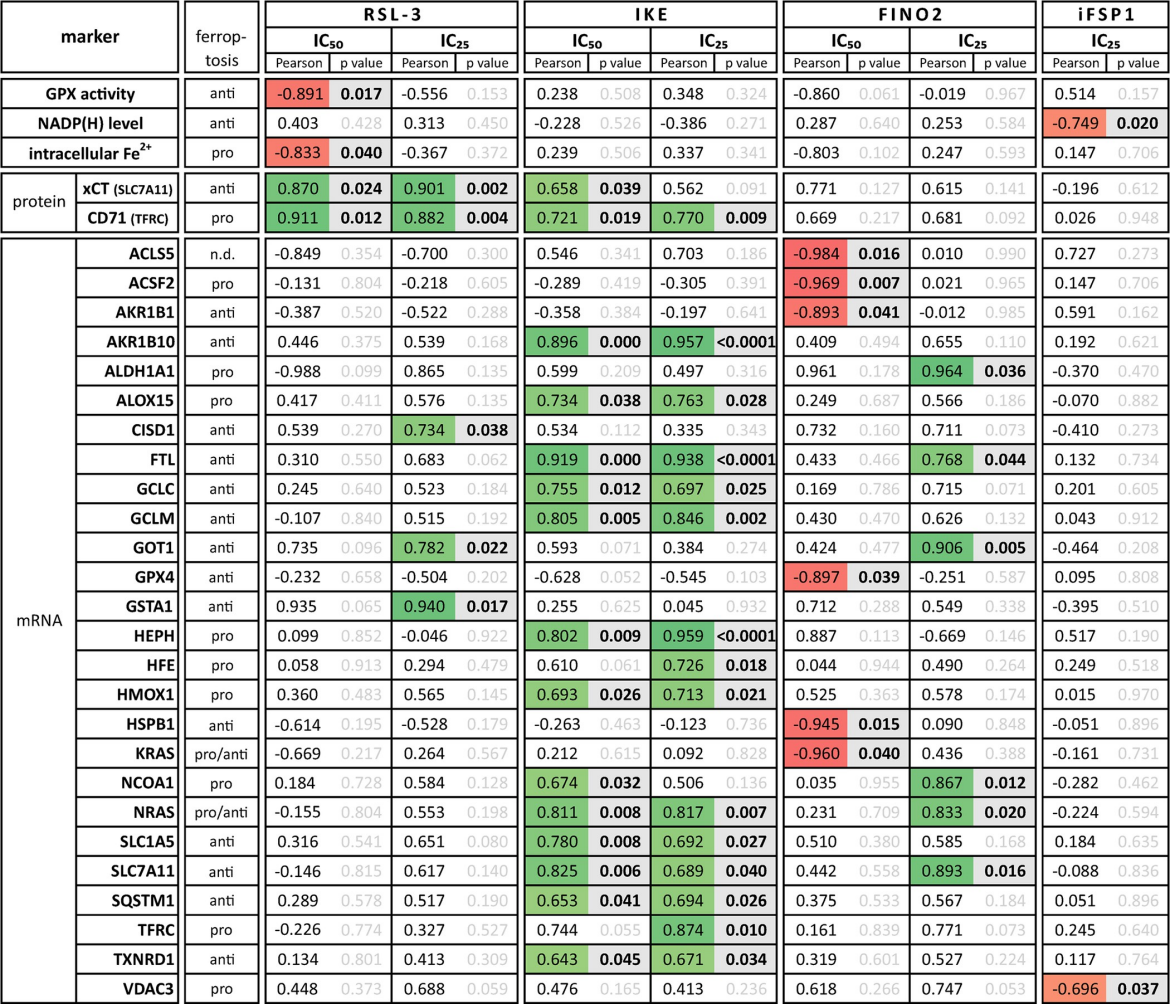
CD71 and SLC7A11 protein expressions significantly correlate with IC_50_ values of IKE and RSL3 in BTC cell lines. Correlation analysis between the IC_25/50_ values of FINs and ferroptosis associated genes/markers. Green boxes indicate a positive correlation, red boxes indicate a negative correlation, bold fond shows a significance correlation (p < 0.05).

As depicted in Fig 5 (shown are only markers, that displayed a significant correlation), we found several significant correlations between the sensitivity of BTC cells and ferroptosis-related markers. Protein and mRNA expression of both, xCT (SLC7A11) and CD71 (TFRC) showed a significant positive correlation with the IC_25/50_ values of RSL3 and IKE. Additionally, in relation to the IC_50_ value of FINO2 and the mRNA expression, only negative correlations were observed.

**Fig 6:**
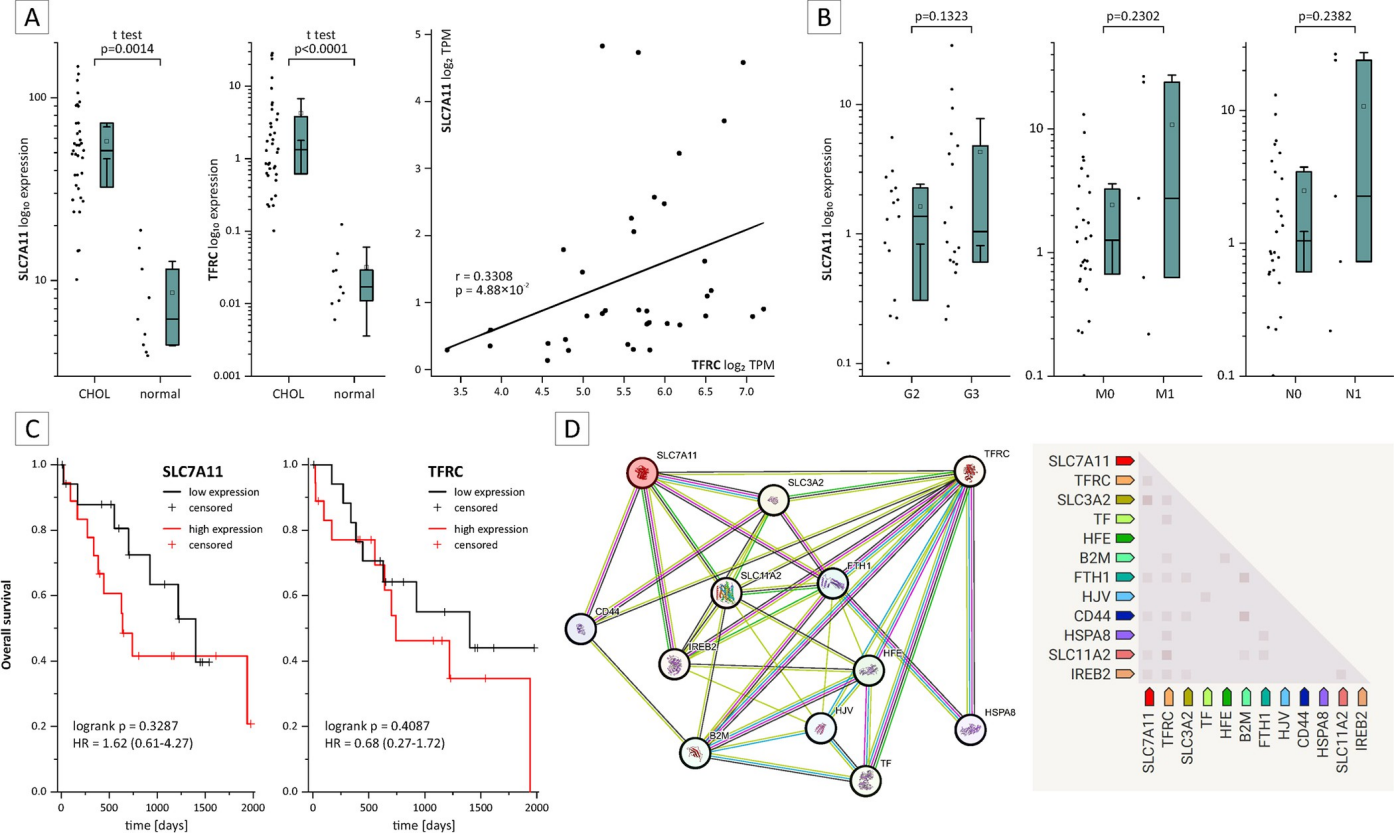
In silico analysis of CD71 and SCL7A11 expression in BTC patients. The results of the different in silico analyses (A to C with OncoDb and UALCAN as well as D with STRING (last accessed 2023-10-10)) showed a significant upregulation of CD71 (TFRC) and SLC7A11 in BTC samples compared to control tissue with a positive correlation between them (A). Furthermore, the expression of SLCA11 was not significantly associated with worse clinicopathological characteristics such as grading, disease status and outcome compared to CD71 (B (CD71 not shown) and C). Protein-protein interaction analysis identified non-ferroptosis beta-2-microglobulin (B2M), CD44 antigen and heat shock protein 8 (HSPA8) as possible interactors between SLC7A11 and CD71, in addition to other "classical" proteins of the iron and/or ferroptosis-related pathways (D). Abbreviations: B2M: Beta-2-microglobulin, CD44: CD44 antigen, FTH1: Ferritin heavy chain 1, HFE: High FE2+ (alias hereditary hemochromatosis protein), HJV: Hemojuvelin, HSPA8: Heat shock 70 kDa protein 8, IREB2: Iron-responsive element-binding protein 2, SLC11A2: Solute carrier family 11 member 2 (alias natural resistance-associated macrophage protein 2), SLC3A2: Solute carrier family 3 member 2 (alias 4F2 cell-surface antigen heavy chain), SLC7A11: solute carrier family 7 member 11 (alias cystine/glutamate transporter), TF: Transferrin and TFRC: Transferrin receptor (alias CD71).

To further observe a clinico-pathological relevance of CD71 and SLC7A11 in BTC patients we performed in silico analysis. In silico gene expression analysis of CD71 and SLC7A11 showed significantly higher expression in tumor tissue compared to normal control tissue and correlation analysis also showed a significant linear positive correlation between CD71 and SLC7A11 (see Fig 6A). Looking in detail, SCL7A11 is more, but not significant linked to grading and tumor progression (nodal and organ metastasis) than CD71 (see Fig 6B, data for CD71 are not shown). Relating to the outcome of patients with BTCs (see Fig 6C), a higher gene expression of CD71 was observed more frequently in BTC cases with better outcome, whereas higher gene expression of SLC7A11 was observed in BTC cases with worse outcome (not significant; see Fig 6C). Examination of protein-protein interactions revealed a more indirect interaction between CD71 and SLC7A11 (see Fig 6D), with this in silico investigation highlighting Beta-2-microglobulin (B2M), CD44-antigen and Heat shock 70 kDa protein 8 (HSPA8) as a possible interactor between SLC7A11 and CD71 in addition to the other “classical” proteins of the iron- and/or ferroptosis related pathways.

## Discussion

BTC is a fatal disease with limited therapeutic options and difficult clinical management due to development of resistance to commonly used chemotherapeutics such as cisplatin that primarily induce apoptosis [3, 4]. Therefore, novel therapeutic strategies that are not solely dependent on the induction of apoptosis are highly attractive as they harbor the potential to circumvent commonly observed resistance mechanisms [42]. In our study, we have investigated the potential of ferroptosis as a non-apoptotic form of cell death as an anti-BTC strategy. Using a comprehensive in vitro model, we were able to demonstrate that various classes of FINs indeed induce ferroptosis in BTC cell lines and exhibit anti-tumor effects in a drug- as well as cell line-specific manner.

We here demonstrate that FINs FINO2, IKE, and RSL3 reduce cell viability in a broad range of BTC cell lines, which is consistent with previous findings in other cancer entities such as colorectal cancer, diffuse large b-cell lymphoma (DLBCL) and fibrosarcoma cells [43–45]. For example, Sui and colleagues demonstrated that treatment with 10 μM RSL3 for 48 hours resulted in the strongest attenuation of cell viability in colon cancer cell lines HCT116, LoVo, and HT29, (IC_50_ values: 2.75 μM-12.38 μM), which is consistent with our findings (IC_50_ values: 0.9–7.9 μM) [44]. Additionally, Gaschler et al. demonstrated, that 10 μM FINO2 had the most potent effect on the reduction of cell viability in the fibrosarcoma cell line HT-1080 [43]. In their research using IKE (a chemical analog of class I FIN erastin), Zhang et al. investigated its effect on cell viability in a subset of 18 different DLBCL cell lines [45]. After treating the cells with IKE for 24 h (starting concentration: 100 μM), they found a heterogeneous sensitivity profile among the cell lines, with IC_50_ values ranging from 0.001 to 97.010 μM [45]. This is comparable to our findings, in the sense that we also found different sensitivities within our in vitro model with IC_50_ values ranging from 3.2 to 50 μM [45]. However, compared to results in studies using bladder cancer and glioblastoma cells, we found that BTC cells were less sensitive as cell viability was only reduced in a subset of tested cell lines using relatively high concentrations of FIN 56 (40 μM) [46, 47]. In the study by Sun et al., FIN56 treatment decreased cell viability in J82, RT-112, T24, and 253J bladder cancer cell lines at lower concentrations, with IC_50_ values ranging from 0.05 to 2.22 μM [46]. Similarly, cell viability of glioblastoma cell lines LN229 and U118 were affected by FIN56 treatment with IC_50_ values of 2.6–4.2 μM, as shown by Zhang et al [47]. A potential explanation for this observation could be a cancer-type specific effect. Furthermore, the precise degradation mechanism of GPX4 by FIN56 remains largely unknown, particularly in BTC [46]. Future studies should investigate this mechanism further [46].

Similarly, treatment with brequinar and iFSP1 did not result in a significant cytotoxic effect in BTC cells. This aligns with previous studies that found pancreatic cancer (10 μM iFSP1) and fibrosarcoma (>100μM brequinar) cell lines to have low sensitivity towards these compounds [48, 49]. A possible explanation might be based on the mode of action of these two FINs: DHODH, the target of brequinar, as well as FSP1, the target of iFSP1, function as cellular protection mechanisms localized in the mitochondria (DHODH) and plasma membrane (FSP1), respectively. They work alongside and independent of GPX4 to assist cells in detoxifying lipid peroxides [48–50]. These mechanisms play a crucial role in maintaining cellular health and preventing oxidative damage. If one protective system, such as FSP1 or DHODH, is inhibited, the cells may rely more heavily on the other protective system [48–50]. This has been demonstrated by recent studies, which described increased cytotoxic effects caused by combined brequinar/iFSP1 and RSL3 treatment [48–50]. For instance, Mao et al. demonstrated, that the combination of brequinar and RSL3 resulted in a stronger reduction of cell viability in HT-1080 cells compared to RSL3 or brequinar only [48]. A similar effect was shown by Yoshioka et al., with iFSP1 and RSL3 in PANC-1 cells [49].

Complete or partial reversal of cell death induced by FINs by different cell death inhibitors can aid to determine the role of ferroptosis in the decrease of cell viability [9]. Stockwell et al. explain that in order to confirm ferroptosis as the (main) cause of cell death, markers of iron-dependent lipid peroxidation must be present [9]. This indicates that lipid peroxidation, which relies on iron, is associated with ferroptotic cell death [9]. Therefore, an antioxidant as well as an iron chelator should be employed. Furthermore, elevated levels of Fe^2+^ and lipid ROS should be observed in case of ferroptosis induction [9].

Our study reveals that the decline in cell viability induced by FINs can be partially reversed by deferoxamine mesylate and ferrostatin-1, which suggests the involvement of ferroptosis. However, we also observed partial reversal of cytotoxicity caused by FINs when BTC cells were co-treated with inhibitors of apoptosis or necroptosis, although the effect was generally less prominent compared to the antioxidant and the iron chelator. Likewise, as shown in other studies, cell death induced by FINs can be partially inhibited by different types of cell death inhibitors with different magnitude. For instance, reduction of cell viability caused by RSL3 in A549 cells could be partially reversed by necrostatin-1 [51]. This is in line with our results, as we also found that necrostatin-1 had positive effects on the cell viability of BTC cells when combined with FINs. Interestingly, Stockwell et al. described that treatment of cells with high concentrations of necrostatin-1 results in unspecific inhibition of ferroptosis, which further underlines the complex interplay between different cell death forms and the difficulty to distinguish them [9]. Similarly, Yuk et al. could demonstrate in hepatocellular carcinoma cell lines, that cell death that was induced by erastin could be reversed by necrostatin-1 [52]. A possible molecular explanation for this observation was, that that the xCT expression, a negative regulator of ferroptosis, was upregulated, following necrostatin-1 treatment [52]. Interestingly, necrostatin-1 was found to inhibit RIPK1-mediated apoptosis in rats with skeletal muscle ischemia [53]. Although necrostatin-1 is a well-established substance to evaluate the involvement of necroptosis following treatment with FINs [9], the additional potential necrostatin-1-based inhibition of apoptosis should be taken into consideration while data interpretation. However, regarding our study, this dual role of necrostatin-1 does not contradict the observed induction of ferroptosis following FIN treatment. Furthermore, as also observed in our study, cell death caused by treatment with FINs was partially reversible by the apoptosis inhibitor ZVAD-FMK. In this regard, Sun et al. have shown that erastin, the precursor of IKE, reduces cell viability through induction of both, ferroptosis and apoptosis in HGC-27 gastric cancer cells [41]. Sun and colleagues measured elevated levels of reactive oxygen species (ROS), a hallmark of ferroptosis, as well as elevated expression of cleaved caspase-3 and PARP proteins, which are hallmarks of apoptosis [41]. Our data indicate that, depending on the substance, ferroptosis is not the sole form of cell death that contributes to decreasing BTC cell viability after treatment with FINs. However, in BTC cells, ferroptosis still represents the primary type of cell death, as indicated by heightened intracellular Fe^2+^ and lipid ROS levels, which are established and characteristic hallmarks of ferroptosis induction [9].

Previously, it was demonstrated that ferroptosis-related genes and markers have been associated with cancer patient prognosis and clinical outcome [54]. For BTC, the expression of ferroptosis-related genes was positively correlated with a poor prognosis of CCA patients [54]. Thus, we investigated whether the susceptibility of BTC cells towards FINs is correlated with established ferroptosis markers.

We found that CD71 and SLC7A11 protein expression were significantly positively correlated with the IC_25_/IC_50_ values of RSL3 and IKE. CD71, also known as transferrin receptor 1, is involved in the import of iron into cells, contributing to the intracellular labile iron pool, which is essential for induction of ferroptosis [12, 55] Interestingly, CD71 is overexpressed in different types of cancer and associated with poor prognosis and survival of cancer patients [56]. On the molecular level, this is explained by the increased demand of cancer cells for iron, which is in turn required for their specific metabolism as well as for proliferation [56]. We therefore also observed CD71 expression in biliary tract cancer patients via in silico analysis and this analysis revealed a significant upregulation of CD71 in BTC tumor tissue. Additionally, clinico-pathological analysis indicated a more “protective” feature of CD71 in BTC.

Regarding ferroptosis, CD71 has been observed to be upregulated in ferroptotic processes [57]. Hiromatsu et al. found that upregulated CD71 expression sensitized hepatocellular carcinoma cell lines to ferroptosis-inducing substances, including artesunate and sorafenib [58]. Furthermore, Stockwell et al. noted that overexpression of CD71 and its localization in the plasma is a characteristic for ferroptosis [9].

Our findings contradict previous results as we detected heightened susceptibility towards RSL3 and IKE in BTC cell lines with low CD71 expression. One explanation might be a tumor type specific effect underlying this observation which needs to be further investigated in future studies.

On a clinicopathological level, SLC7A11 has been observed to be overexpressed in various types of cancer [59]. Lin et al. conducted a study that identified overexpression of SLC7A11 in more than 20 cancer entities and patients in this state were associated with a poor prognosis [59]. The in silico analysis also revealed an upregulation of SLC7A11 in BTC patients, which was associated with higher pathological tumor grading, disease progression and worse outcome, in contrast to the in silico findings for CD71. Interestingly, protein-protein interaction analysis revealed a relationship between the non-iron and non-ferroptosis related proteins B2M, CD44 and HSPA8 with SLC7A11 and CD71, with CD44 appearing to play an important role in carcinogenesis and prognosis in BTC [60] and to be an interesting target alone [61] or in combination with a ferroptosis inhibitor as recently shown [62]. SLC7A11 is a negative regulator of ferroptosis, and its higher expression is associated with ferroptosis resistance [63–65]. For instance, SLC7A11 deletion in pancreatic ductal adenocarcinoma cell lines MiaPaCa-2 and Capan-2 resulted in induction of ferroptosis and attenuation of cell viability, as shown by Daher et al. [64]. Additionally, in mouse embryogenic fibroblasts, cell viability reduction caused by erastin treatment was attenuated, if SLC7A11 was overexpressed compared to wild-type cells [66]. This is in accordance with our observation, as high SLC7A11 expression correlates with a decreased sensitivity of BTC cells towards IKE and RSL3 treatment.

## Conclusion

This study investigates the effects and consequences of established ferroptosis-inducing drugs in BTC. We discovered that different types of FINs caused cell death in a cell line—and substance-specific manner that is accompanied by increased Fe^2+^ and lipid ROS and could be significantly inhibited by DFX and Fer-1. Additionally, our results indicate that the sensitivity of BTC cells to IKE and RSL3 is positively associated with the expression of CD71 and SLC7A11 proteins that, therefore, might serve as response-predictive biomarkers. These findings suggest that inducing ferroptosis may present a promising therapeutic intervention for biliary tract cancer and warrants further investigation through in vitro and in vivo studies.

## Supporting information

S1 Fig Viability of HeLa and Hep-G2 cells is attenuated after treatment with FINs. Cells were incubated for 48 h with a 10-step 1:2 dilution series starting with 10 μM brequinar, 40 μM FIN56, 10 μM FINO2, 50 μM iFSP1, 50 μM IKE and 10 μM RSL3. (A) Cell viability data of HeLa and Hep-G2 after FINs treatment. Data is related to untreated control cells (UTC) and shown as mean values +/- SEM of n = 3 biological replicates. (B) Heatmap of calculated IC_25/50_ values (via four-parameter logistic expression) of FINs in HeLa and Hep-G2 cell lines; green: low IC_25/50_ values, red high IC_25/50_ values.
(PDF)

S2 Fig Cell morphology is affected by FINs in selected BTC cell lines. Cell morphology pictures under a light microscope (10X) of selected BTC cell lines CCC-5, HuH-28 and KKU-055 following FINs treatment for 48h; UTC = untreated control.
(PDF)

S3 Fig Reduction of cell viability by FINs is partially reversed by deferoxamine mesylate and ferrostatin-1. Cells were treated with 1 μM brequinar, 10 μM FINO2, 50 μM iFSP1, 50 μM IKE and 10 μM RSL3 with/or only 20 μM of deferoxamine, ferrostatin-1, necrostatin-1 and Z-VAD-FMK. (A) Cell viability data is shown as mean values +/- SEM of n = 3 biological replicates of HeLa cells with FINs (light grey) and FINs + cell death inhibitors (dark grey) for 24 h. (B) Cell viability data is shown as mean values +/- SEM of n = 3 biological replicates of Hep-G2 cells with FINs (light grey) and FINs + cell death inhibitors (dark grey) for 24 h. (C) Cell viability data is shown as mean values +/- SEM of n = 3 biological replicates of selected cell lines after treatment with cell death inhibitors only.
(PDF)

S4 Fig Baseline protein expression of ACSL4, CD71, FTH1, GPX4 and xCT; GPX-activity, intracellular iron levels and NADP(H) concentration in HeLa and Hep-G2 cell lines. (A) Baseline protein expression of ACSL4, CD71, FTH1, GPX4 and xCT in HeLa and Hep-G2 cells with representative western blot images. Shown are data as mean values +/- SEM of n = 3 biological replicates relative to β-actin expression. (B) Baseline GPX-activity of HeLa and Hep-G2 cells. Data is shown as mean +/- SEM of n = 3 biological replicates. (C) Baseline intracellular iron levels of HeLa and Hep-G2 cells. Data is shown as mean +/- SEM of n = 3 biological replicates. (D) Baseline NADP(H) concentration in HeLa and Hep-G2 cells. Data is shown as mean +/- SEM of n = 3 biological replicates.
(PDF)

S5 Fig Baseline mRNA expression (2-ΔCt) of ferroptosis-related genes in BTC cell lines. Obtained Ct values were related to a virtual reference gene (see material and methods). n = 1 biological replicate. The following genes could not be detected in any cell line: BBC3, HAMP, LOX, MAP1LC3A, NFE2L2, NOX4, PCBP1, SLC11A2, SLC3A2 and TFR2. CS, CHAC1, HMOX2 were excluded because of amplification in non-template control. mRNA expression was compared between cell lines within a gene: from red = lowest expression to green = highest expression.
(PDF)

S1 Raw images (PDF)
