# Supplementary material for: The efficacy of ferroptosis-inducing compounds IKE and RSL3 correlates with the expression of ferroptotic pathway regulators CD71 and SLC7A11 in biliary tract cancer cells
Source: PLoS One. 2024 Apr 11;19(4):e0302050. doi: 10.1371/journal.pone.0302050 (PMC11008848; doi:10.1371/journal.pone.0302050)
Supplement: S1 Fig — Cells were incubated for 48 h with a 10-step 1:2 dilution series starting with 10 μM brequinar, 40 μM FIN56, 10 μM FINO2, 50 μM iFSP1, 50 μM IKE and 10 μM RSL3. (A) Cell viability data of HeLa and Hep-G2 after FINs treatment. Data is related to untreated control cells (UTC) and shown as mean values +/- SEM of n = 3 biological replicates. (B) Heatmap of calculated IC25/50 values (via four-parameter logistic expression) of FINs in HeLa and Hep-G2 cell lines; green: low IC25/50 values, red high IC25/50 values. (PDF) [file pone.0302050.s001.pdf]

A

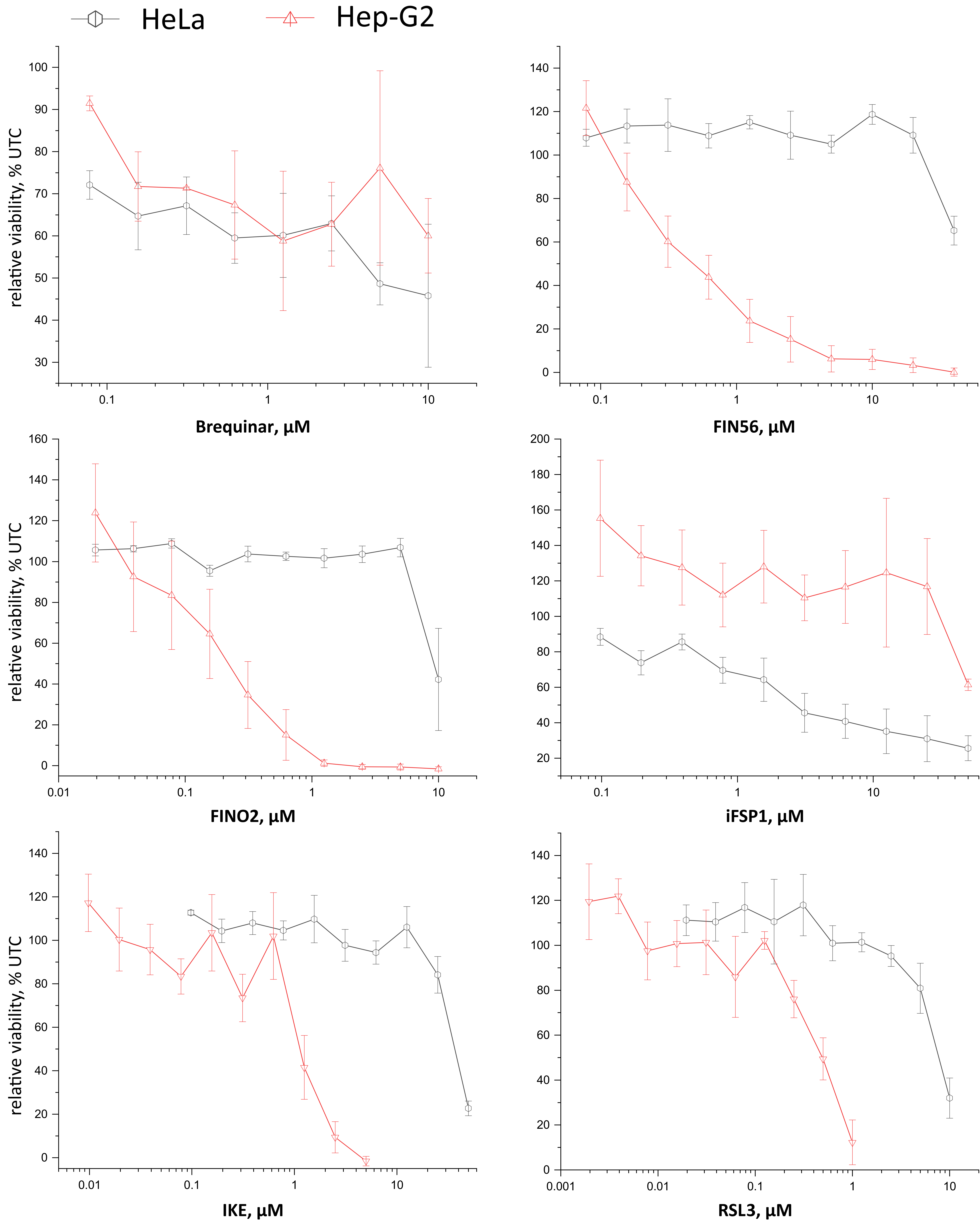

B

IC$_{50}$ (µM)

	Brequinar	FIN56	FINO2	iFSP1	IKE	RSL3
HeLa	-	-	-	3.02	36.52	8.06
Hep-G2	-	0.44	0.21	-	14.67	0.47

IC$_{25}$ (µM)

	Brequinar	FIN56	FINO2	iFSP1	IKE	RSL3
HeLa	-	24.17	5.03	0.71	23.14	8.06
Hep-G2	-	0.22	0.13	46.55	0.70	0.29
