# Supplementary figures and images for: The efficacy of ferroptosis-inducing compounds IKE and RSL3 correlates with the expression of ferroptotic pathway regulators CD71 and SLC7A11 in biliary tract cancer cells
Source: PLoS One. 2024 Apr 11;19(4):e0302050. doi: 10.1371/journal.pone.0302050 (PMC11008848; doi:10.1371/journal.pone.0302050)

CCC-5

HuH-28

KKU-055

UTC

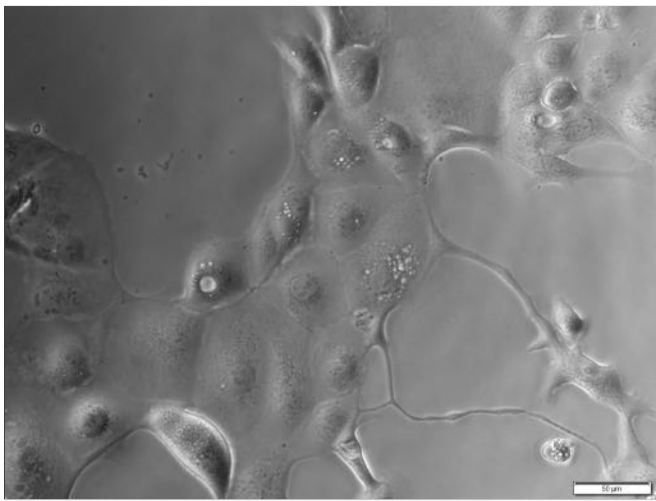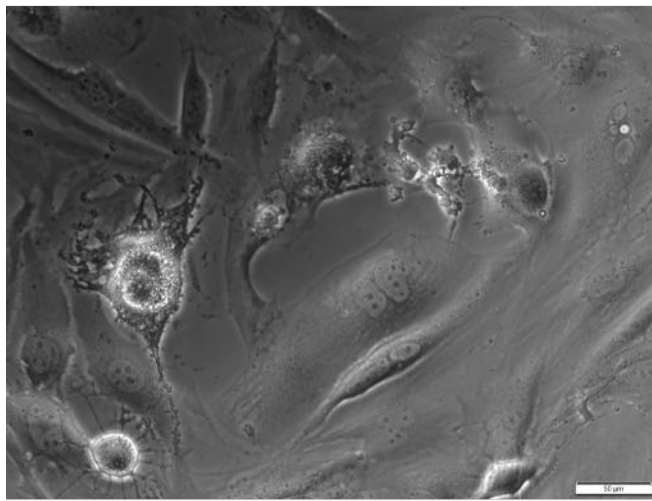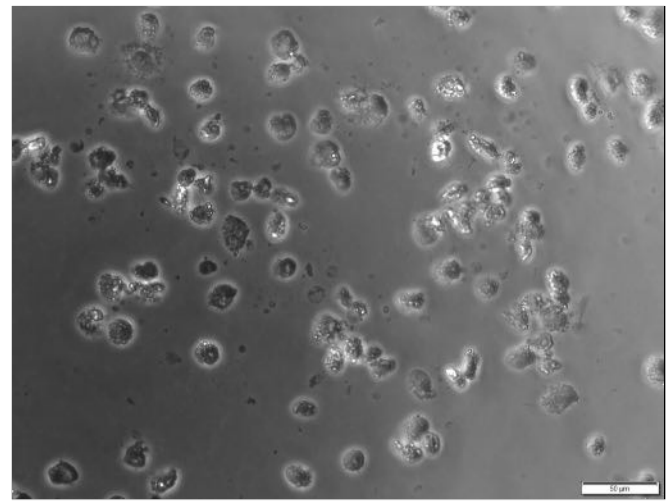

Brequinar

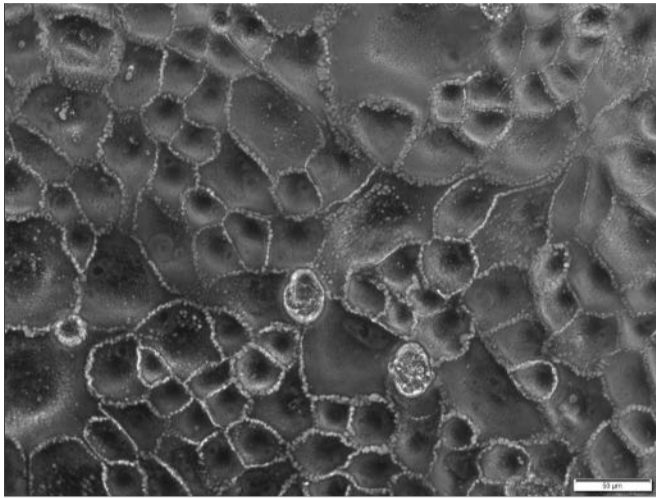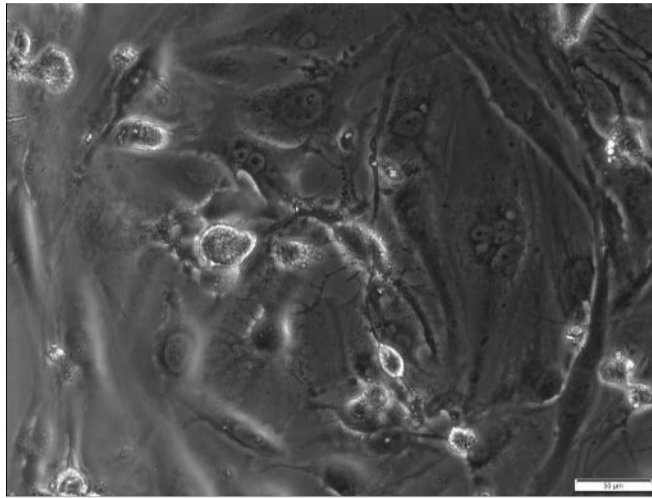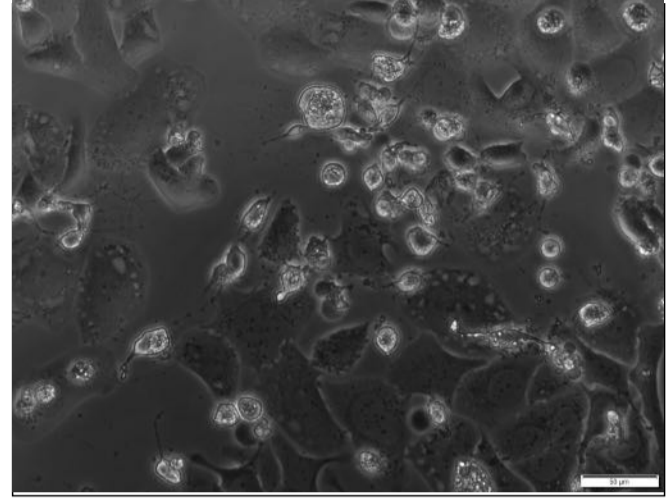

FIN56

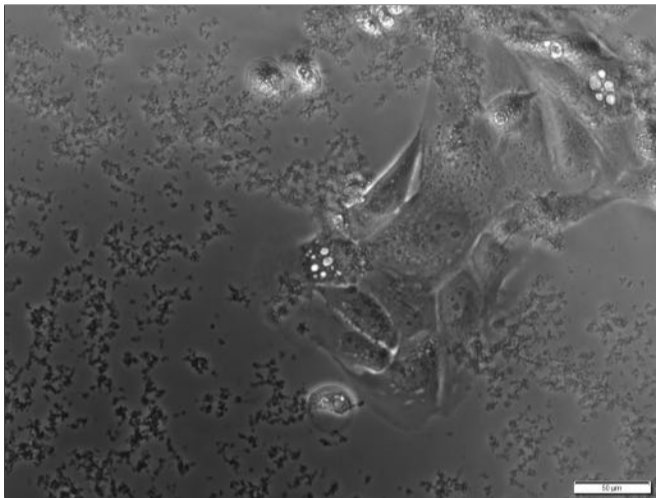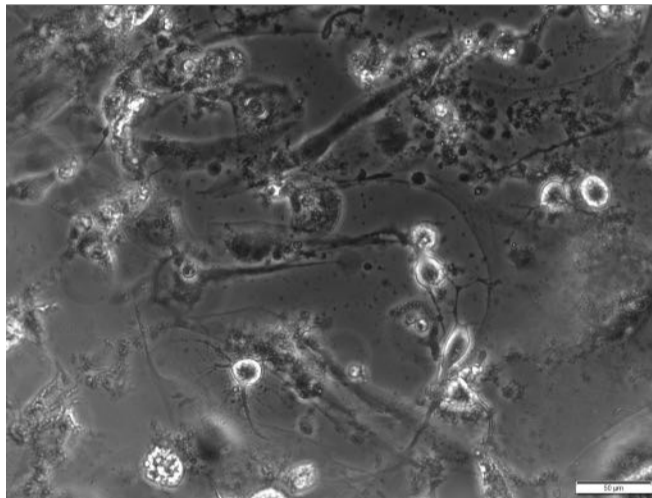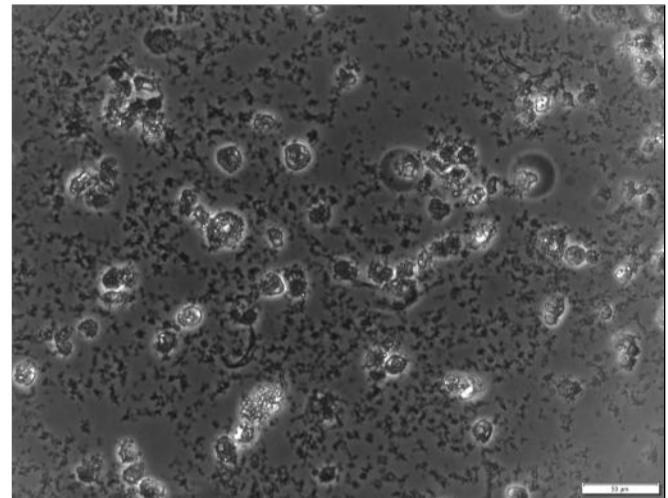

FINO2

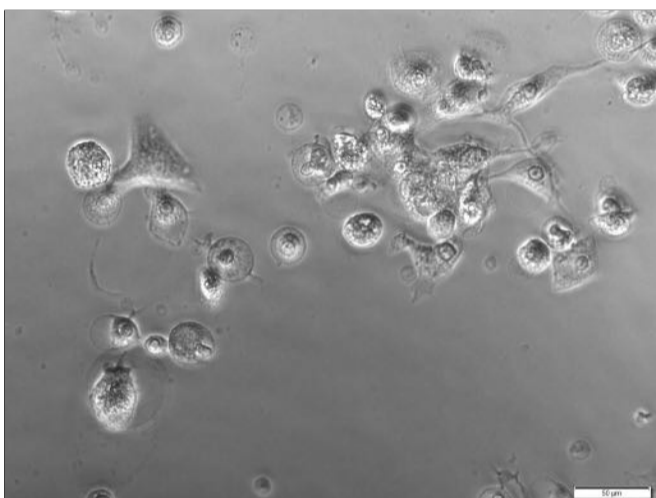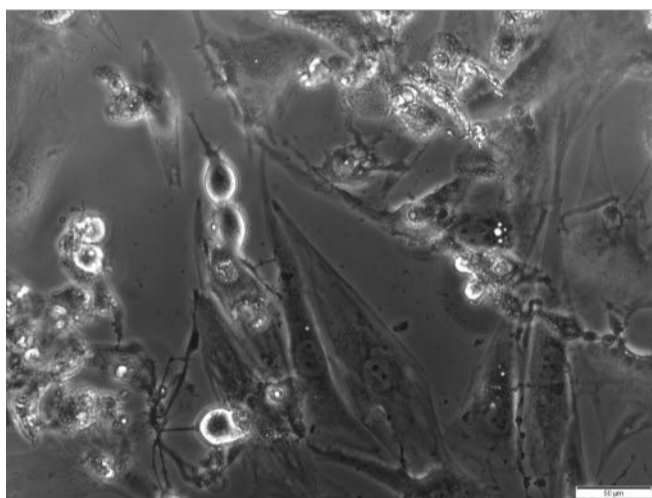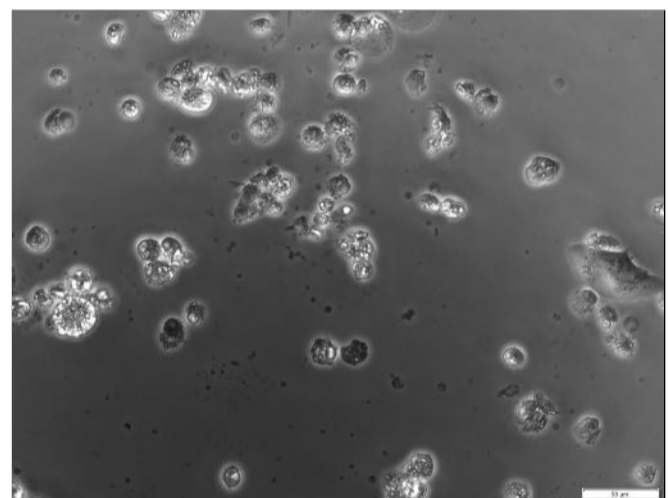

iFSP1

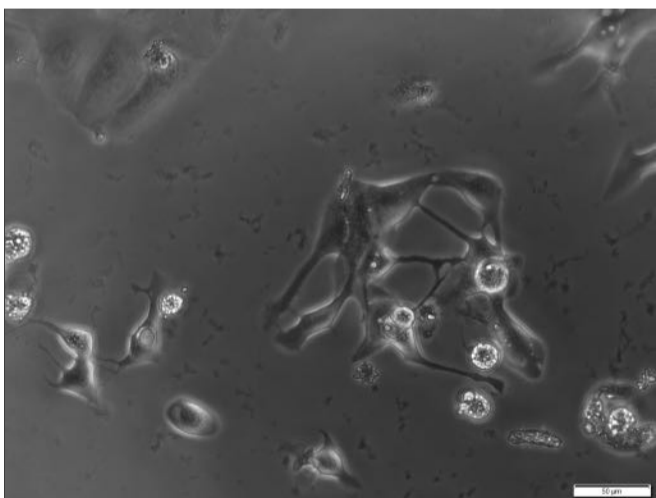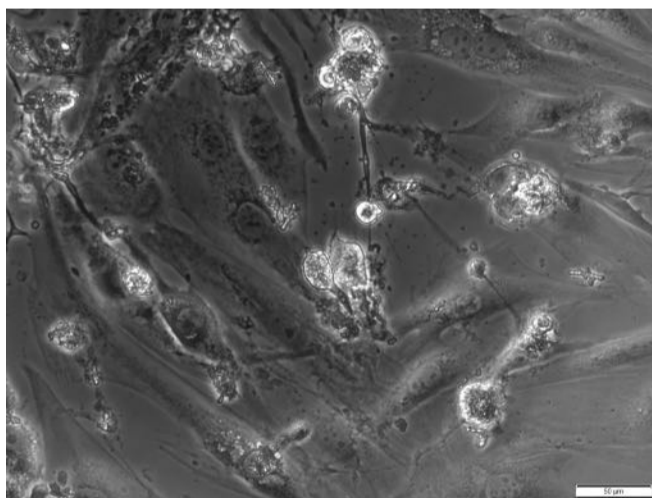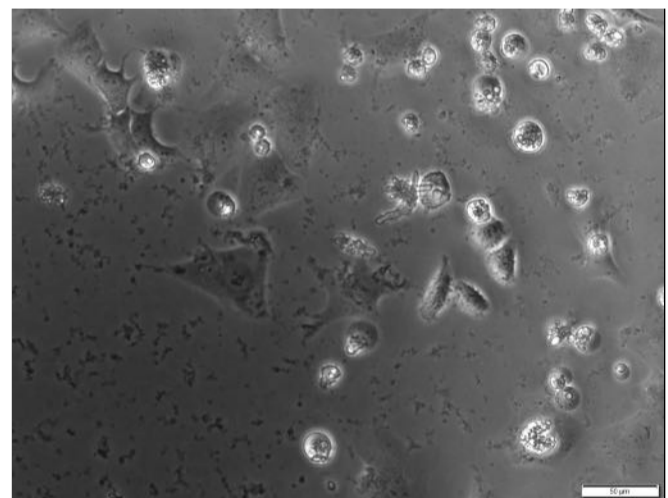

IKE

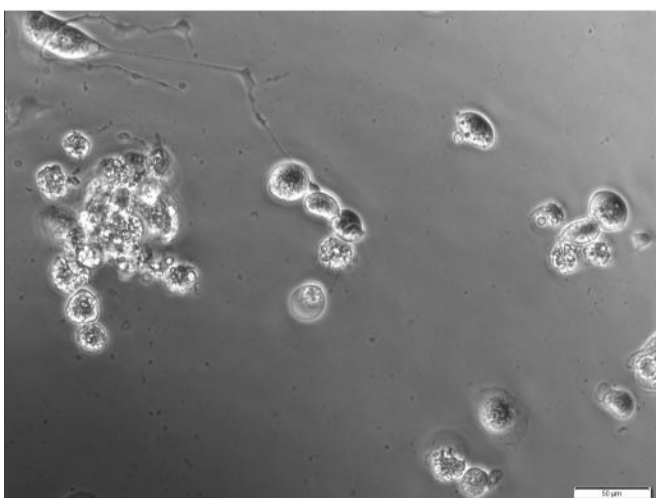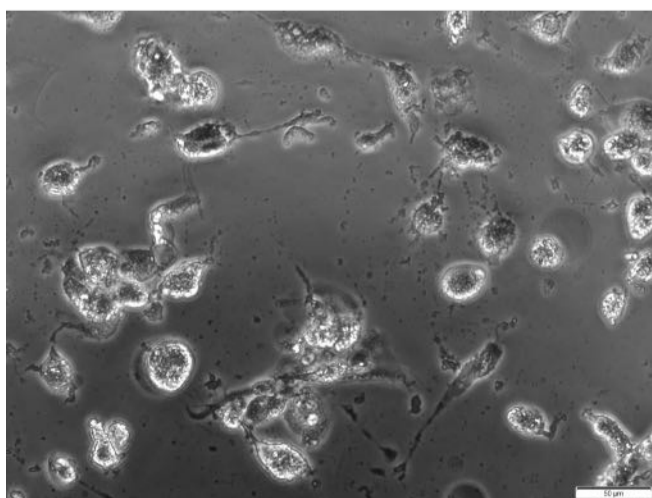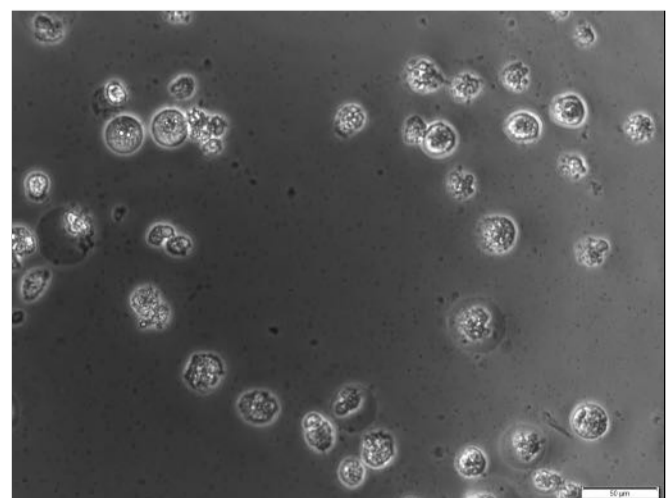

RSL3

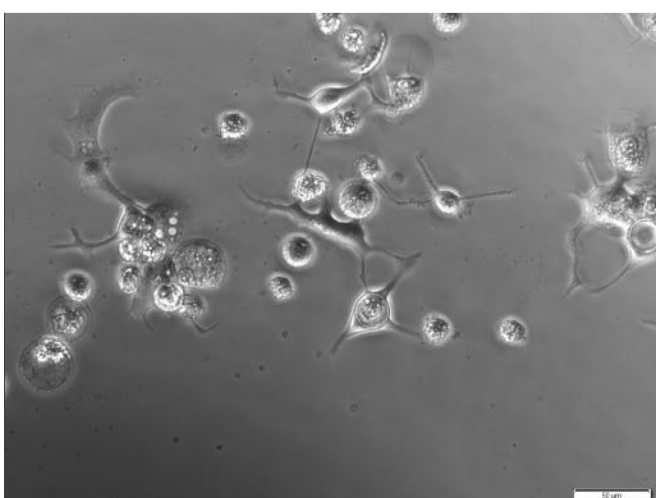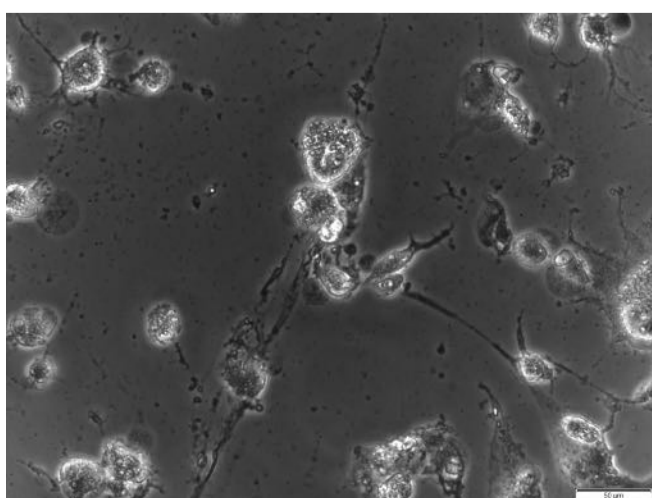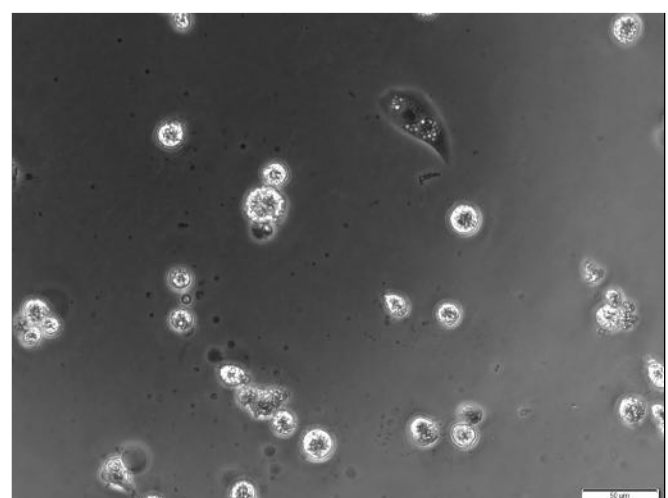

Supplement: S2 Fig — Cell morphology pictures under a light microscope (10X) of selected BTC cell lines CCC-5, HuH-28 and KKU-055 following FINs treatment for 48h; UTC = untreated control. (PDF) [file pone.0302050.s002.pdf]

A

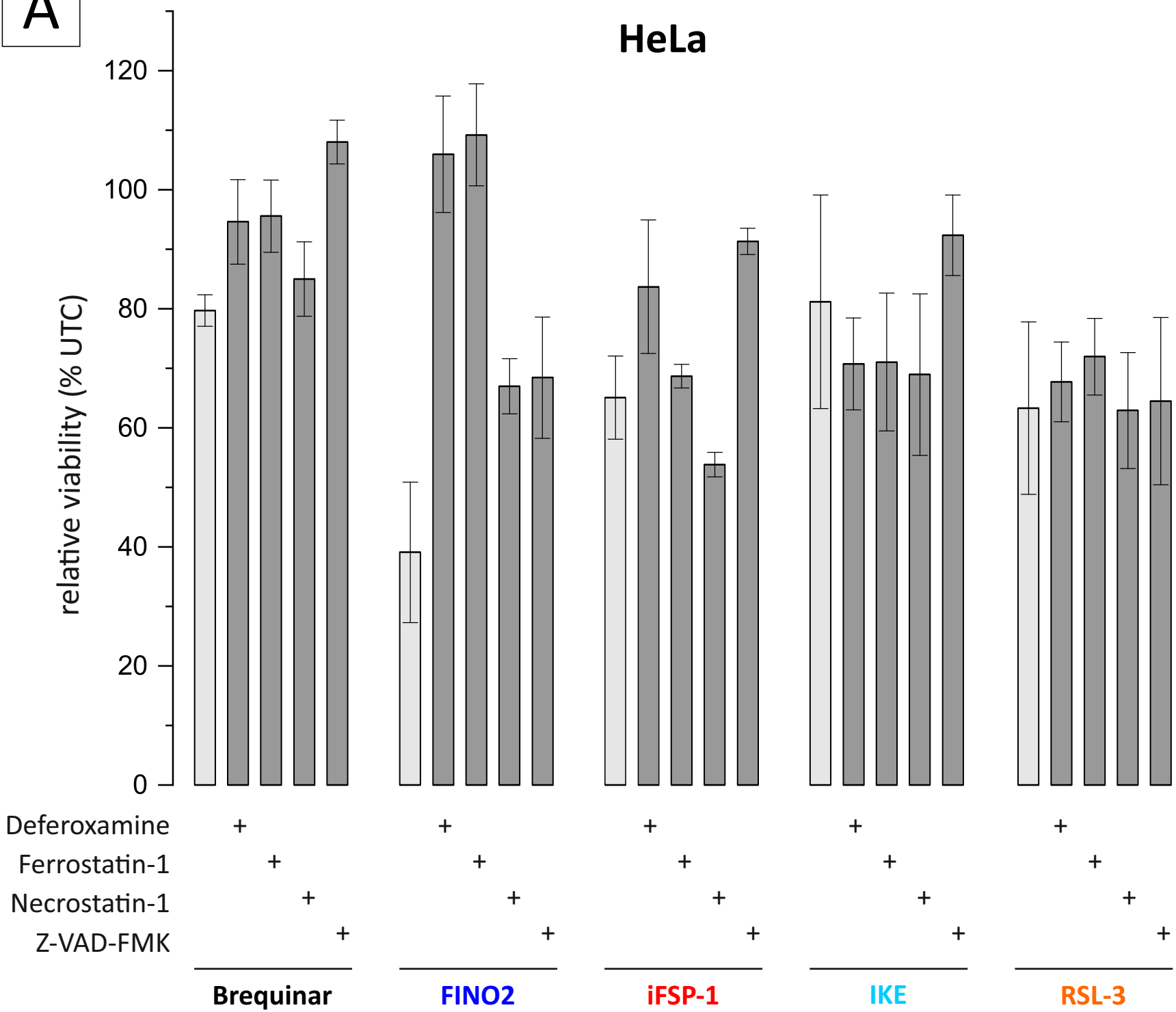

B

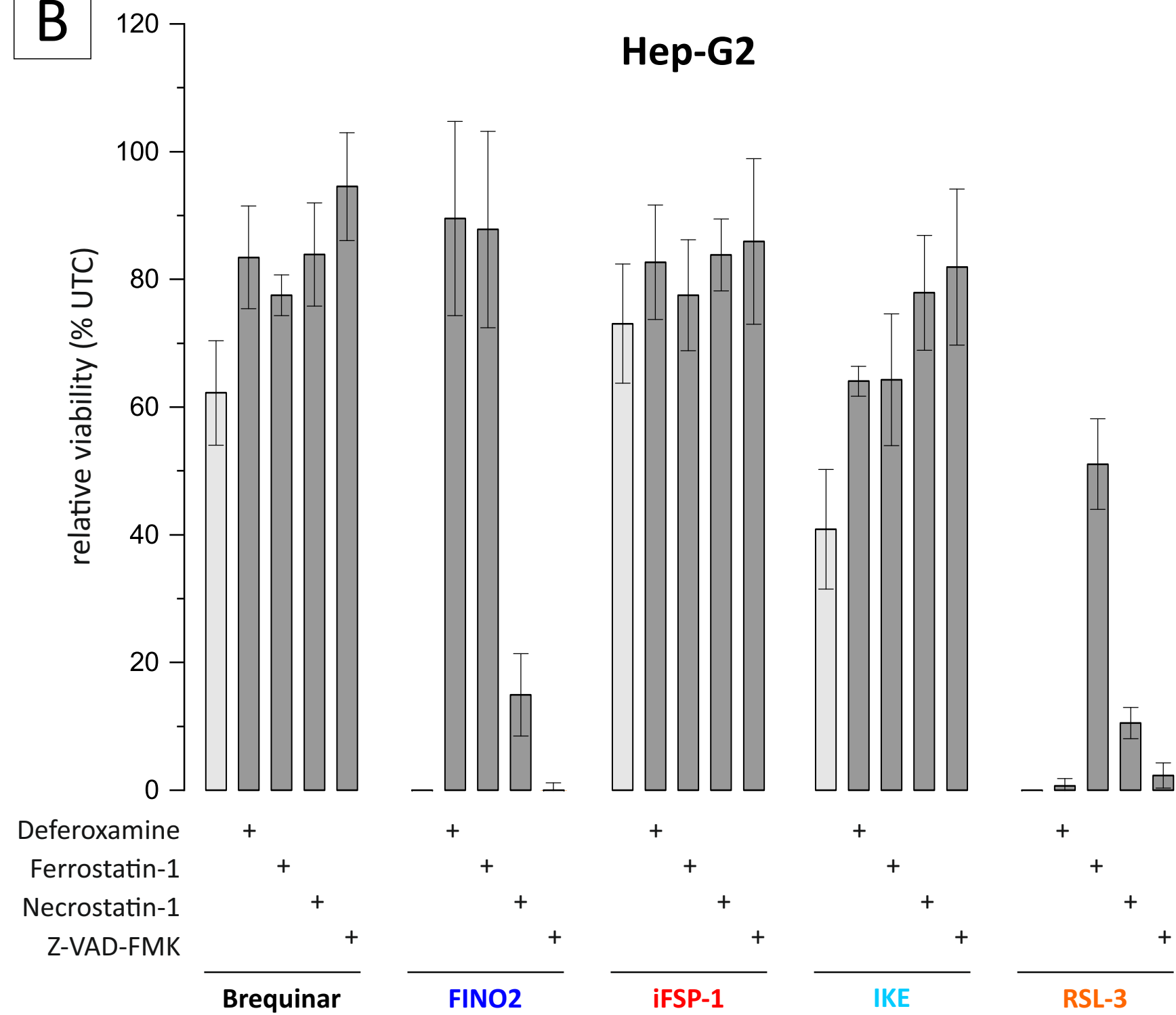

C

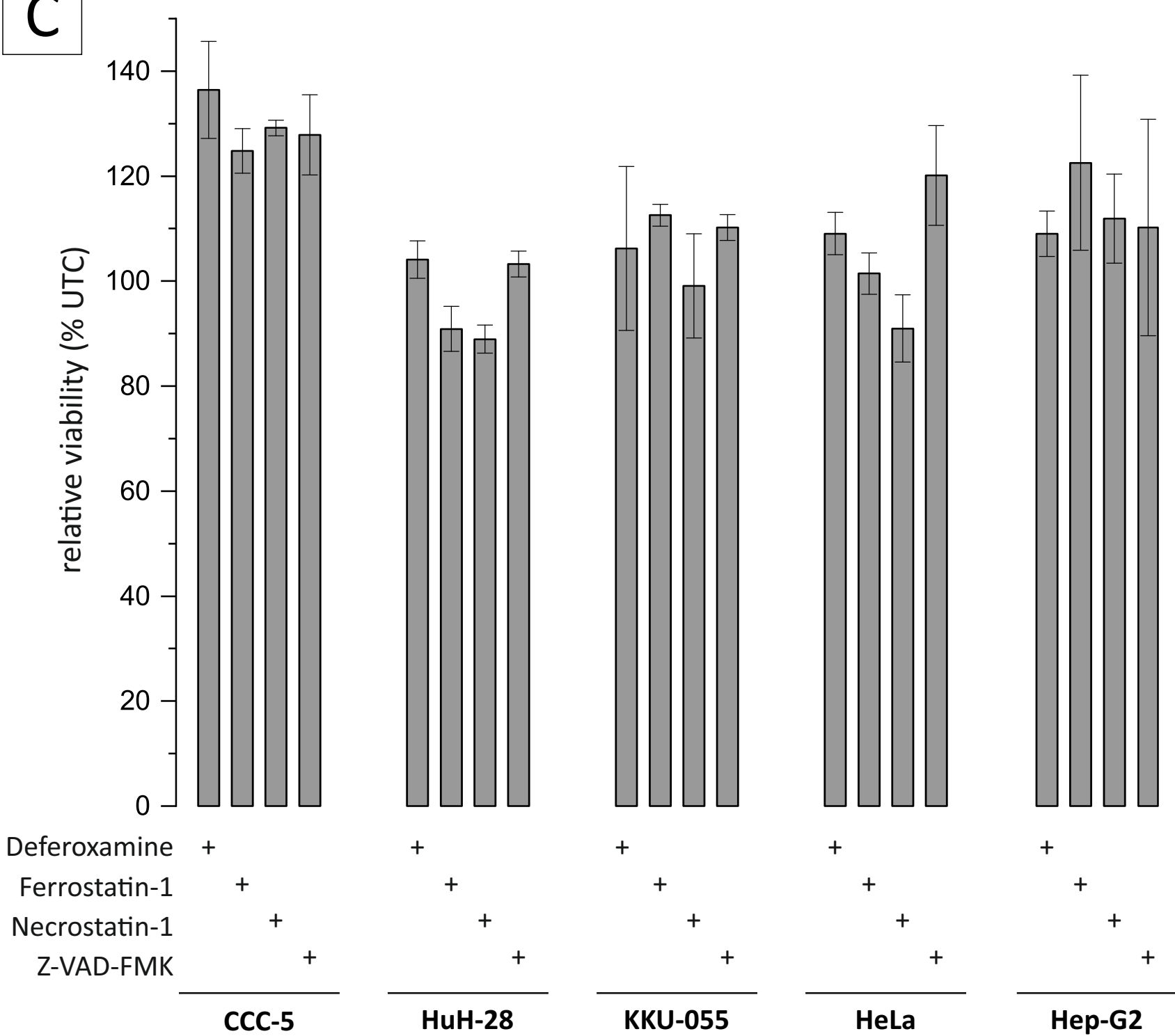

Supplement: S3 Fig — Cells were treated with 1 μM brequinar, 10 μM FINO2, 50 μM iFSP1, 50 μM IKE and 10 μM RSL3 with/or only 20 μM of deferoxamine, ferrostatin-1, necrostatin-1 and Z-VAD-FMK. (A) Cell viability data is shown as mean values +/- SEM of n = 3 biological replicates of HeLa cells with FINs (light grey) and FINs + cell death inhibitors (dark grey) for 24 h. (B) Cell viability data is shown as mean values +/- SEM of n = 3 biological replicates of Hep-G2 cells with FINs (light grey) and FINs + cell death inhibitors (dark grey) for 24 h. (C) Cell viability data is shown as mean values +/- SEM of n = 3 biological replicates of selected cell lines after treatment with cell death inhibitors only. (PDF) [file pone.0302050.s003.pdf]

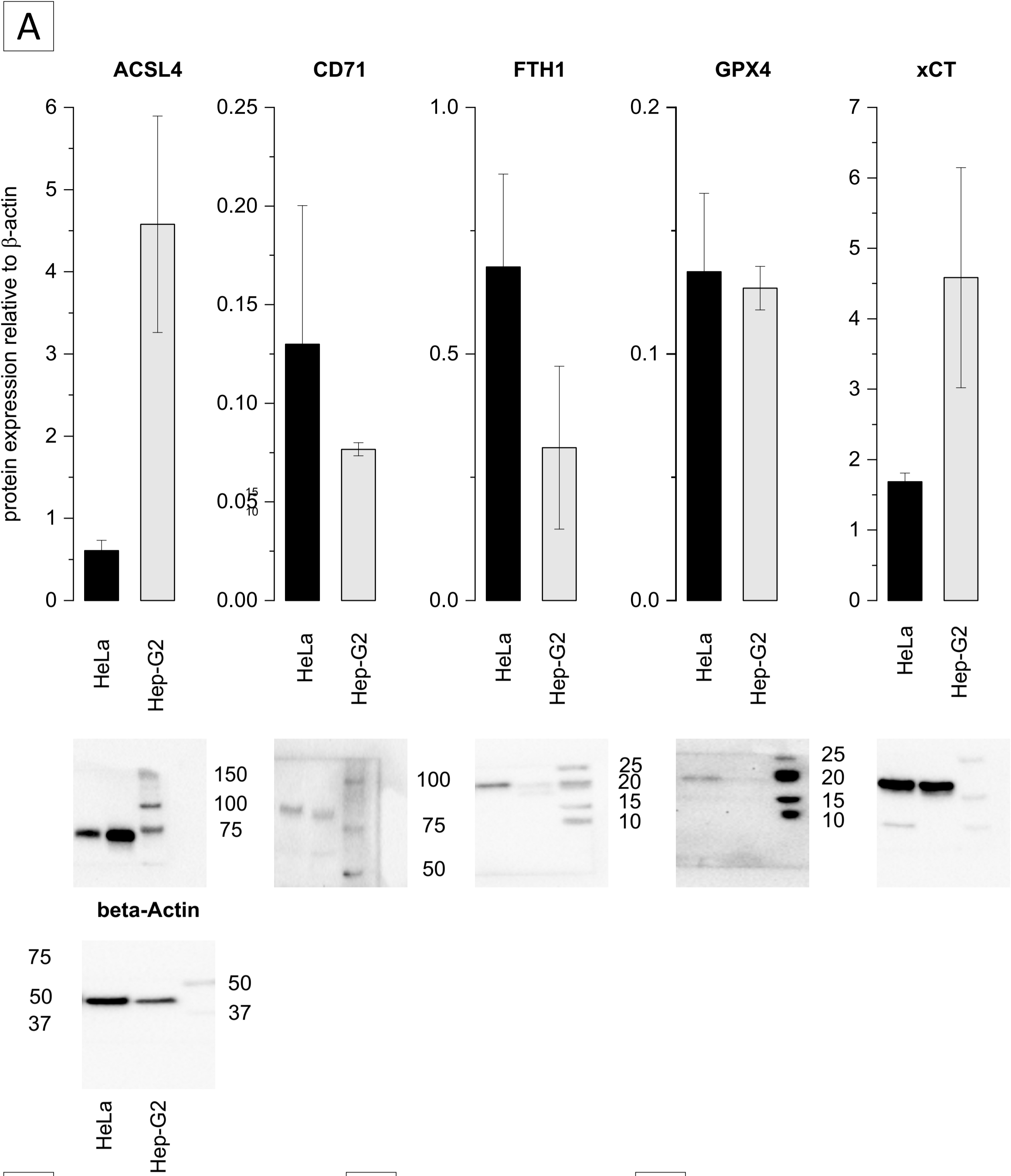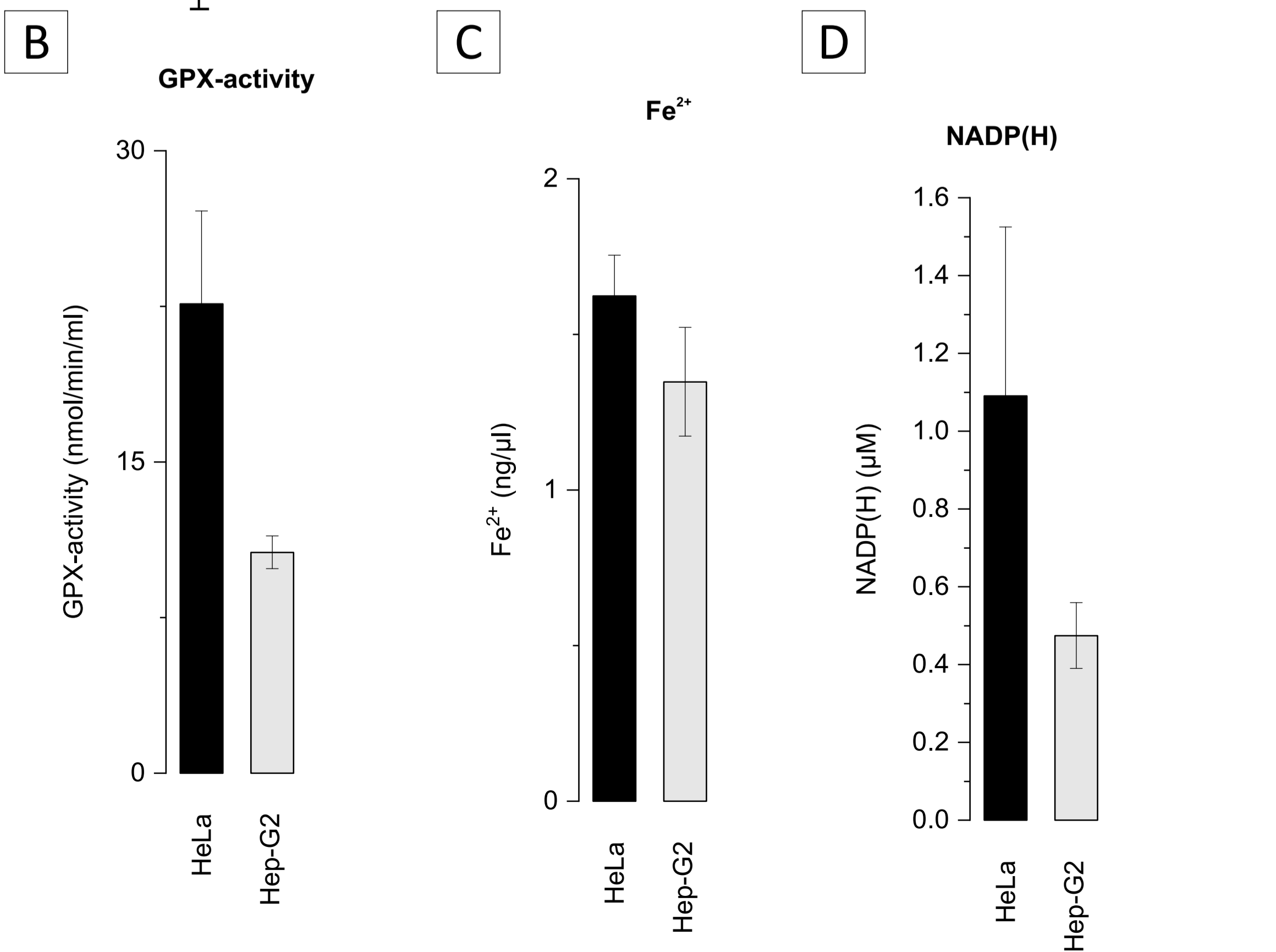

Supplement: S4 Fig — (A) Baseline protein expression of ACSL4, CD71, FTH1, GPX4 and xCT in HeLa and Hep-G2 cells with representative western blot images. Shown are data as mean values +/- SEM of n = 3 biological replicates relative to β-actin expression. (B) Baseline GPX-activity of HeLa and Hep-G2 cells. Data is shown as mean +/- SEM of n = 3 biological replicates. (C) Baseline intracellular iron levels of HeLa and Hep-G2 cells. Data is shown as mean +/- SEM of n = 3 biological replicates. (D) Baseline NADP(H) concentration in HeLa and Hep-G2 cells. Data is shown as mean +/- SEM of n = 3 biological replicates. (PDF) [file pone.0302050.s004.pdf]
