# Supplementary material for: The efficacy of ferroptosis-inducing compounds IKE and RSL3 correlates with the expression of ferroptotic pathway regulators CD71 and SLC7A11 in biliary tract cancer cells
Source: PLoS One. 2024 Apr 11;19(4):e0302050. doi: 10.1371/journal.pone.0302050 (PMC11008848; doi:10.1371/journal.pone.0302050)
Supplement: S5 Fig — Obtained Ct values were related to a virtual reference gene (see material and methods). n = 1 biological replicate. The following genes could not be detected in any cell line: BBC3, HAMP, LOX, MAP1LC3A, NFE2L2, NOX4, PCBP1, SLC11A2, SLC3A2 and TFR2. CS, CHAC1, HMOX2 were excluded because of amplification in non-template control. mRNA expression was compared between cell lines within a gene: from red = lowest expression to green = highest expression. (PDF) [file pone.0302050.s005.pdf]

Genes of interest	Cell lines									
	CCC-5	EGI-1	HuCCT-1	HUH-28	KKU-055	KKU-100	NOZ	OCUG-1	OZ	TFK-1
ACLS4	4.97E-03				1.18E-02	1.02E-02	1.81E-03	3.06E-03	1.53E-02	2.58E-03
ACLS5	6.76E-02				1.79E-05	1.07E-03		2.09E-01		7.91E-03
ACSF2	1.44E-02	1.36E-02	1.52E-03	4.24E-03	8.09E-04	1.52E-03	6.93E-03	1.18E-03	5.28E-03	2.75E-03
ACSL1				5.79E-05	9.56E-06					
ACSL3	1.18E-02	6.39E-03	1.21E-03	2.91E-03	8.27E-03	1.24E-02	3.75E-04	2.86E-03	8.13E-03	1.23E-02
ACSL6		1.87E-05			1.24E-05		1.09E-05		4.23E-06	
AKR1B1	2.45E+01		4.87E-01	2.69E+00	1.30E+00	7.77E+00		4.62E+00	4.09E-02	1.24E+00
AKR1B10	1.65E-03	2.69E-03	1.57E-02	2.69E-03	9.87E-05	7.51E-03	4.59E-04	7.62E+00	1.20E+00	7.64E+00
AKR1C1	1.28E-03									
ALDH1A1		5.23E-04			4.56E-03	1.84E-03		1.80E-01	7.30E-04	1.94E+00
ALOX15	1.27E-04	4.72E-04	6.20E-05	2.47E-05	1.19E-05	2.28E-06	6.61E-05			6.77E-04
ATG5	6.37E-03		3.92E-03	2.59E-03	1.34E-03			1.09E-02	3.11E-03	3.07E-03
ATG7	1.80E-03		1.31E-03	6.29E-04		4.48E-03			3.46E-03	1.21E-03
ATP5G3	1.23E-02	1.18E-02	5.77E-03	5.51E-03	6.85E-03	1.37E-02	3.06E-03	1.25E-02	3.29E-02	1.79E-02
BRAF	1.00E-02	5.20E-03	3.47E-03	1.72E-03	7.43E-03	7.67E-04	6.11E-03	5.89E-03	4.02E-03	6.68E-03
CARS1	7.89E-02	9.12E-02	1.63E-02	9.13E-02	7.85E-02	5.71E-02	1.96E-01	6.15E-02	8.38E-02	5.44E-02
CISD1	1.71E-03	6.26E-03	3.63E-03	2.37E-03	9.32E-03	5.94E-03	6.82E-03	4.45E-03	1.21E-02	1.54E-02
CP	4.43E-04	7.68E-06	2.17E-05	5.10E-06	2.82E-03	8.72E-03	1.62E-05	5.76E-06		3.27E-03
CYBA	3.03E-06		1.42E-06				5.61E-06		7.25E-06	4.81E-06
CYBB		2.78E-06								
EMC2	5.27E-02	7.03E-02	4.38E-02		7.91E-02	5.73E-02	4.34E-02	3.50E-02	7.29E-02	5.28E-02
EPRS									6.36E-04	2.46E-03
FTH1	6.70E-03	6.66E-02	1.88E-02	5.53E-02	5.00E-03	6.31E-02	3.44E-02	7.64E-02	4.06E-03	9.15E-02
FTL	1.64E+00	7.25E-01	3.22E+00	1.63E+00	8.28E-02	4.38E+00	8.55E+00	1.82E+01	3.82E+00	1.89E+01
FTMT	4.16E-06	4.86E-04		1.54E-04	2.12E-04		8.28E-06		1.15E-05	7.12E-06
GCLC	4.20E-02	2.15E-02	1.74E-02	2.27E-03	1.16E-02	4.19E-02	2.68E-02	7.47E-02	9.45E-02	8.37E-02
GCLM	1.42E-02	4.69E-03	1.43E-02	1.56E-02	1.41E-02	2.43E-02	8.34E-03	1.42E-01	1.41E-02	2.24E-01
GOT1	9.40E-02	1.67E-01	1.44E-01	1.34E-02	1.30E-01	8.70E-02	6.04E-01	1.33E-01	2.13E-01	6.14E-01
GPX4	8.35E-02	5.98E-02	3.66E-02	4.48E-02	3.39E-02	5.27E-02	3.34E-02	1.94E-02	3.18E-02	3.82E-02
GSL2	1.23E-04	2.63E-03	4.81E-04		4.59E-04		3.05E-03	2.45E-04	1.49E-03	2.87E-04
GSS	1.19E-01	1.27E-01	9.59E-02	7.39E-02	6.80E-02	1.30E-01	2.54E-01	1.61E-01	1.75E-01	1.00E-01
GSTA1	4.46E-05	1.61E-04		6.12E-05	7.38E-05			4.79E-05		1.64E-04
GSTP1	1.07E-01	1.10E-01	8.61E-02				2.27E-01	1.20E-01	3.13E-01	5.11E-01
HARS			3.56E-04	4.32E-06		1.30E-03	5.50E-04	4.72E-04		1.29E-04
HEPH	3.05E-06	1.22E-04	6.96E-06	1.64E-04	1.30E-04	1.58E-04	6.98E-05	6.50E-03	2.24E-04	
HFE	1.46E-02	3.57E-03	4.52E-03	9.76E-04	8.46E-04	1.43E-02	2.35E-03	2.08E-02	6.63E-03	2.37E-02
HMOX1	7.10E-04	3.98E-04	2.99E-03	4.29E-03	1.69E-04	2.74E-02	6.34E-03	3.86E-02	2.02E-04	8.81E-02

Genes of interest	Cell lines									
	CCC-5	EGI-1	HuCCT-1	HUH-28	KKU-055	KKU-100	NOZ	OCUG-1	OZ	TFK-1
HRAS	3.12E-02	7.11E-02	1.77E-02	3.11E-02	8.32E-02	7.21E-02	2.16E-01	7.35E-02	1.20E-01	2.33E-02
HSF1	2.33E-04	2.75E-05	4.00E-04	1.44E-05	3.32E-05	4.90E-04	6.50E-03	1.09E-03	2.82E-03	1.55E-04
HSPB1	1.76E+00	2.83E-02	1.34E-01	2.95E-01	3.90E-01	2.51E-01	7.10E-01	4.46E-01	3.57E-03	9.48E-02
IREB2	4.49E-03	2.50E-03	1.24E-03	6.77E-04	4.46E-03	8.64E-04	1.04E-03	2.07E-03	6.24E-03	1.33E-02
KEAP1	9.13E-04	4.43E-04	3.00E-04	1.12E-04	4.26E-04	2.30E-03	6.99E-03	1.98E-03	5.41E-03	8.92E-04
KRAS	7.88E-02	5.06E-02	2.42E-02		3.13E-02	1.35E-02	5.01E-01		4.24E-02	2.72E-02
LPCAT3	3.96E-02	1.64E-02	2.95E-02	7.89E-03	5.60E-03	3.87E-02	4.53E-02	2.60E-02	2.07E-02	2.07E-02
MAP1LC3B	9.83E-02	2.46E-03	4.69E-02	9.37E-03	2.67E-02	1.42E-01	1.27E-01	9.52E-02	1.90E-01	2.50E-02
MAP1LC3C	1.59E-05	1.95E-05	4.66E-05	1.02E-05	3.84E-06	2.35E-04		8.28E-05	9.85E-06	1.67E-04
NCOA1	1.06E-01	8.76E-02	9.86E-02	1.57E-02	6.10E-02	2.96E-02	8.34E-02	1.01E-01	1.70E-01	1.91E-01
NOX1	3.05E-04		2.78E-05			2.35E-04	4.07E-05	1.26E-04	3.43E-04	1.35E-04
NOX3							2.45E-05			
NQO1	1.03E-04	1.44E-01			2.15E-01	9.57E-04	2.23E-05	1.62E-05	7.26E-05	2.39E-04
NRAS	2.37E-02	5.37E-03		9.57E-04	1.57E-02	1.19E-02	1.98E-02	4.27E-02	1.79E-02	6.37E-02
PANX1	1.10E-05							8.59E-06		7.42E-06
PANX2	3.12E-03	7.50E-05	5.37E-04	1.84E-04	7.97E-05	1.51E-02	9.32E-05	5.40E-03	6.57E-03	3.22E-02
PCBP2	9.21E-01	1.65E+00	1.14E+00	7.28E-01	2.05E+00	1.07E+00	2.58E+00	6.25E-01	1.69E+00	1.01E+00
PPARG	5.09E-02	1.57E-04	4.81E-03	2.34E-05		9.92E-04	2.20E-02	2.23E-02	1.88E-03	3.94E-03
PRDX6	3.82E-02	2.44E-01	6.01E-02	1.57E-01	9.04E-01	1.32E-02	3.56E-02	4.53E-02	2.84E-02	4.62E-02
PRNP	1.10E-01	7.94E-02	2.46E-01	2.73E-01	7.27E-02	2.07E-01	2.46E-01	2.08E-01	3.64E-01	1.86E-01
PTGES2	1.97E-03		5.70E-05			7.18E-04	2.22E-04	5.13E-04	1.42E-03	3.47E-04
RPL8	4.25E+00	2.47E+00	5.57E+00	7.50E-01	2.14E+00	2.59E+00	1.51E+01	4.78E+00	6.09E+00	2.98E+00
SAT1	7.66E-01	8.14E-02	4.72E-02	1.54E-01	1.61E+00	4.19E+00	2.99E-01	4.52E-01	1.99E+00	3.65E-01
SAT2	2.69E-03	1.81E-04		5.00E-05	1.94E-04	5.16E-03	1.05E-03	1.09E-03	8.19E-04	6.79E-04
SLC1A5	2.18E-03	1.98E-03	3.77E-03	3.30E-03	1.05E-03	6.42E-03	8.95E-03	8.22E-03	6.58E-03	8.34E-03
SLC39A14	2.31E-04	3.97E-05	2.05E-03	1.28E-04	3.70E-04	3.06E-03	2.19E-03	1.50E-03	1.44E-03	3.77E-04
SLC39A8	7.05E-03	1.08E-02	2.02E-02	2.59E-02	4.57E-03	5.07E-02	1.90E-02	2.39E-02	9.52E-03	2.10E-02
SLC40A1	2.03E-03	9.50E-04	6.24E-04	6.85E-04	6.99E-04	1.66E-02		1.04E-03	3.23E-02	2.61E-02
SLC7A11	4.37E-04	3.22E-06	6.61E-05	5.95E-06		3.69E-04	1.92E-03	1.89E-03	2.33E-03	1.79E-03
SQSTM1	1.26E-01	7.36E-03	1.17E-01	1.43E-02	1.32E-03	2.67E-01	7.90E-02	2.94E-01	7.27E-02	5.01E-01
STEAP3	6.02E-04	9.40E-04	4.42E-04	5.21E-04	6.17E-04	1.20E-03	2.63E-03	8.43E-04	2.25E-03	4.05E-04
STIM1	9.95E-06	4.00E-06	8.41E-06			1.38E-05	1.78E-05	1.19E-05	2.86E-05	
TF	6.03E-05				3.39E-06					8.78E-06
TFRC	3.43E-02	4.01E-03		1.35E-03	3.13E-02		2.16E-02	1.07E-01		4.68E-02
TP53	1.18E-05	3.01E-05	4.51E-06	1.01E-05	1.18E-05	4.19E-05	2.44E-05	9.70E-06	4.52E-05	3.08E-05
TXNRD1	5.33E-02	5.81E-05	8.81E-03	1.15E-04	9.36E-05	8.99E-02	6.25E-03	9.18E-02	5.90E-02	1.79E-01
VDAC2	8.18E-01	3.92E-01	9.59E-01	1.30E-01	7.73E-01	1.29E+00	2.74E+00	7.61E-01	1.30E+00	1.01E+00
VDAC3	1.12E-01	1.81E-01	3.38E-01	4.31E-02	3.92E-01	1.67E-01	2.44E-01	2.92E-01	1.31E-01	4.58E-01
