# Supplementary material for: The efficacy of ferroptosis-inducing compounds IKE and RSL3 correlates with the expression of ferroptotic pathway regulators CD71 and SLC7A11 in biliary tract cancer cells
Source: PLoS One. 2024 Apr 11;19(4):e0302050. doi: 10.1371/journal.pone.0302050 (PMC11008848; doi:10.1371/journal.pone.0302050)

Uncropped and unadjusted Western Blot Images for Fig 4A and S4A Fig.
Chemiluminescence was analyzed using the ChemiDoc MP System and Image Lab Software from Biorad.
Grey densities of bands were calculated using ImageJ Software to evaluate protein expression in relation to the loading control beta-Actin. (See Material and Methods)

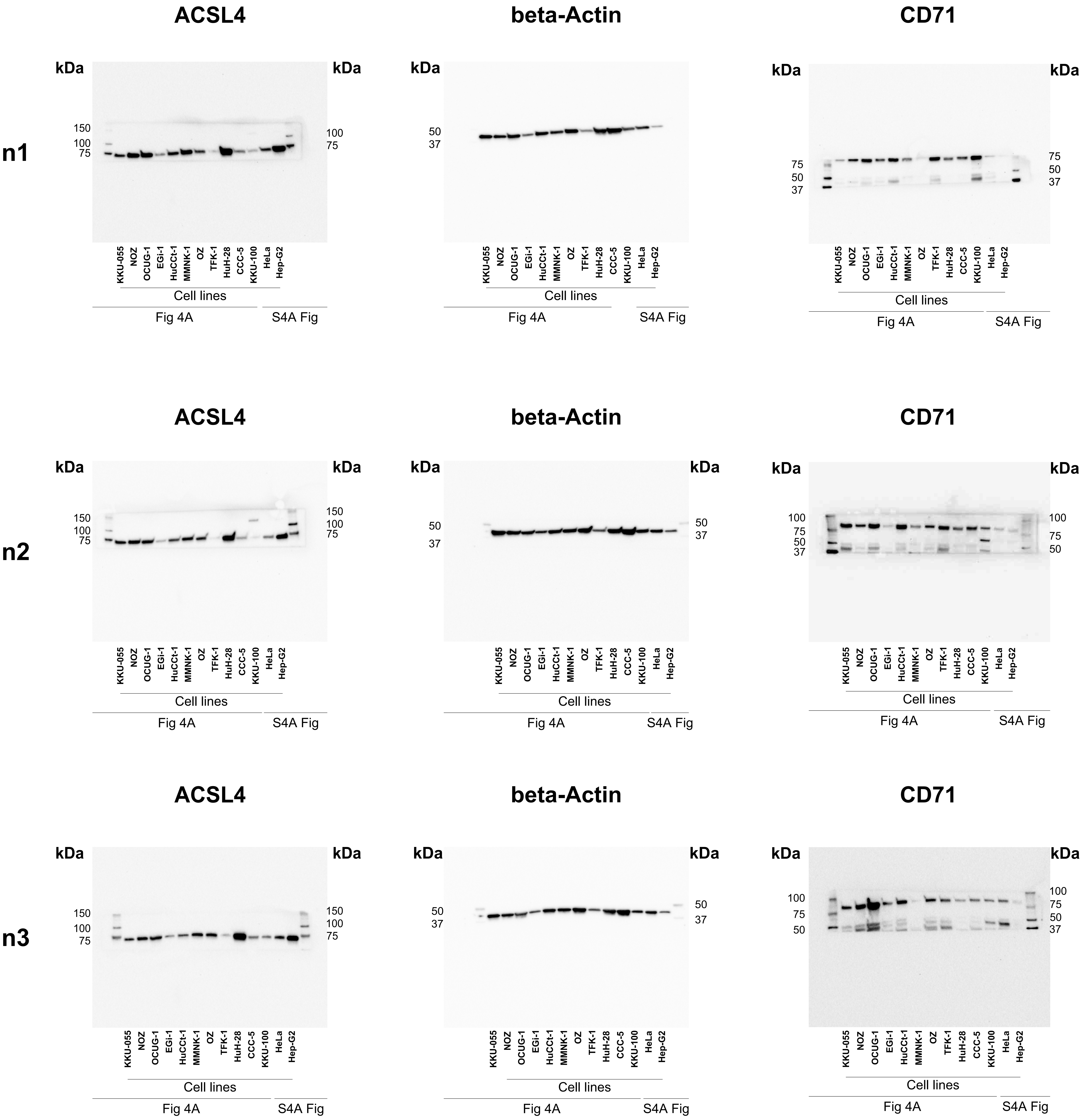

Uncropped and unadjusted Western Blot Images for Fig 4A and S4A Fig.
Chemiluminescence was analyzed using the ChemiDoc MP System and Image Lab Software from Biorad.
Grey densities of bands were calculated using ImageJ Software to evaluate protein expression in relation to the loading control beta-Actin. (See Material and Methods)

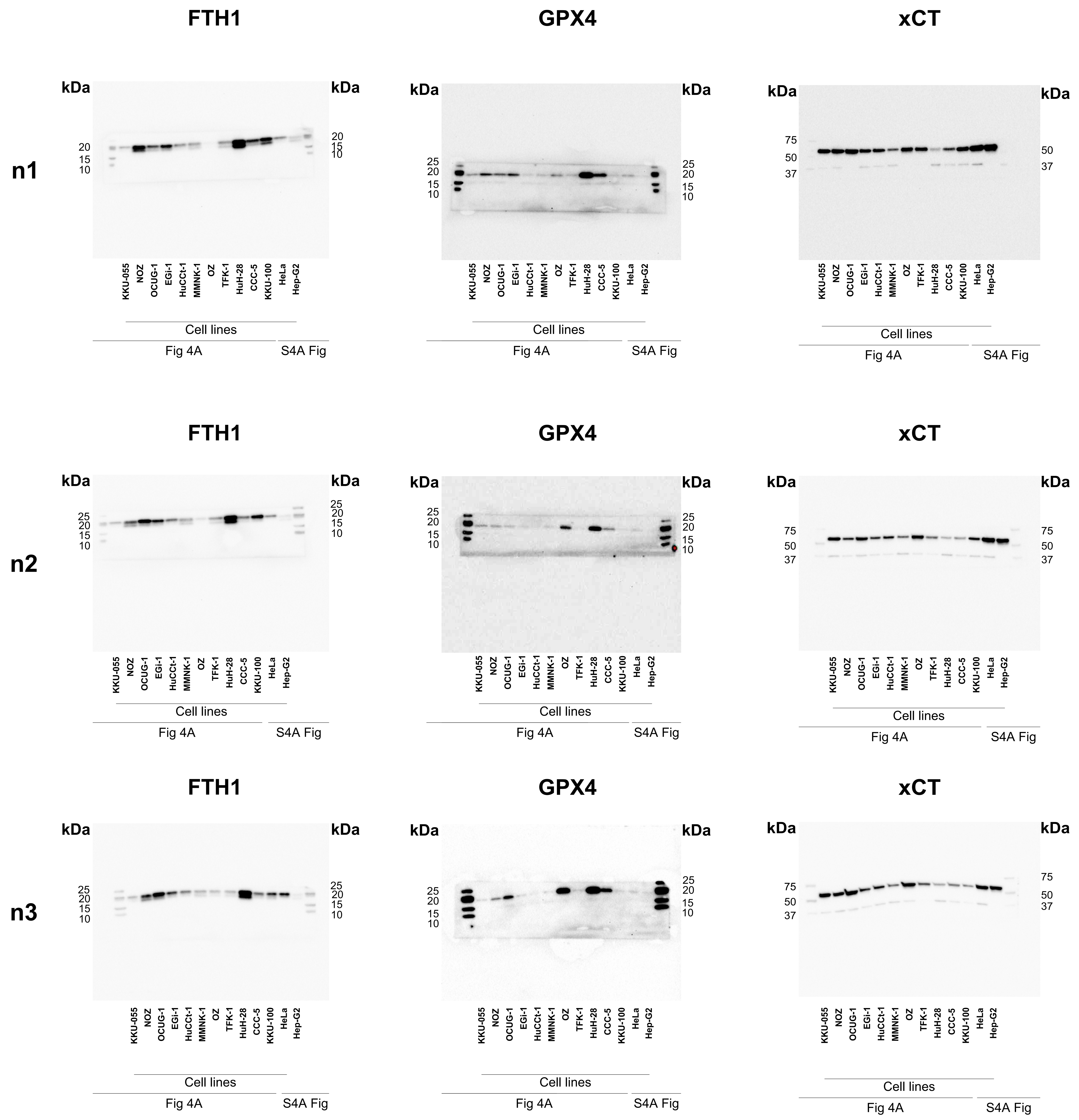

Supplement: S1 Raw images — (PDF) [file pone.0302050.s006.pdf]
